# R&D grants and R&D tax credits to foreign-owned subsidiaries: Does supporting multinational enterprises’ R&D pay off in terms of firm performance improvements for the host economy?

**DOI:** 10.1007/s10961-023-09995-9

**Published:** 2023-02-13

**Authors:** Helena Lenihan, Kevin Mulligan, Justin Doran, Christian Rammer, Olubunmi Ipinnaiye

**Affiliations:** 1 Department of Economics, Kemmy Business School, University of Limerick, Limerick, Ireland; 2 Spatial and Regional Economics Research Centre, Department of Economics, Cork University Business School, University College Cork, Cork, Ireland; 3 Department Economics of Innovation and Industrial Dynamics, ZEW - Leibniz Centre for European Economic Research, Mannheim, Germany

**Keywords:** Public support for R&D, Foreign-owned subsidiaries, Firm performance, Multinational enterprises (MNEs), R&D grants, R&D tax credits, D22, O25, D04, H25, O31

## Abstract

The subsidiaries of foreign-owned multinational firms make significant contributions to national Research and Development (R&D) in many host countries. Policymakers in host countries often support subsidiaries’ R&D efforts, through R&D grants and R&D tax credits. A key objective of this funding is to leverage R&D-driven firm performance benefits for the host economy. However, the subsidiary's parent firm may decide not to commercially exploit the results from host country-funded R&D projects, in the host country. Therefore, supporting subsidiaries’ R&D presents a unique risk, that significant amounts of scarce public R&D funding may translate into little, or no firm performance payoffs for the host economy. To address this issue, we construct a unique panel dataset, containing 24,404 observations of firms in Ireland over a 10-year period. Using this rich data, we first evaluate the impact of R&D grants and R&D tax credits on subsidiaries’ R&D. We then examine the link between policy-induced R&D from each policy instrument, and subsidiaries’ firm performance in the host country. Our study provides the first evaluation of (1) whether public R&D funding stimulates additional R&D investment in subsidiaries, (2) whether policy-induced R&D drives subsidiaries’ firm performance in the host country, and (3) the differential effects of R&D grants and R&D tax credits. We find that both R&D policy instruments drive subsidiary R&D, and that the policy-induced R&D results in substantial host country improvements in turnover, exports, and value added. Our results suggest several policy implications, particularly for economies pursuing an R&D strategy which targets foreign-owned subsidiaries.

**Supplementary Information:**

The online version contains supplementary material available at 10.1007/s10961-023-09995-9.

## Introduction

Firm-level Research and Development (R&D) is a key driver of firm performance and national economic growth (Coccia, 2018; Kancs & Siliverstovs, 2016). The subsidiaries of foreign-owned multinational firms make significant contributions to national R&D in many host countries (Landman et al., 2022; Moncada-Paternò-Castello et al., 2011), particularly in small, open economies (Cunningham et al., 2020; Dachs et al., 2008). Policymakers often pursue an industrial strategy of incentivising foreign-owned subsidiaries to increase their R&D activities, either through direct subsidies or tax incentives, in anticipation of reaping economic gains (Guimón, 2009; OECD, 2015). This study provides, to the best of our knowledge, the first evaluation of whether R&D grants and R&D tax credits drive additional R&D investment in foreign-owned subsidiaries, *and* whether this publicly-funded R&D translates into firm performance improvements for the host economy. Foreign-owned firms may use R&D support from the host country, but commercialise host country-funded R&D results at other locations in their global R&D networks. By focusing on the impact of public R&D funding on foreign-owned subsidiaries, we provide new insights on this important policy issue.

The need for the above research agenda is pressing. Discussing Foreign Direct Investment (FDI) R&D policy, Rama (2008: 353) highlighted that “after decades of debate it is still unclear whether it is desirable for the … host country”. More recently, Landman et al. (2022: 1) have echoed this point, stating that “[d]espite the key role of multinational enterprises (MNEs) in both international markets and domestic economies, there is no consensus on their impact on their host economy”. Moreover, both Guimón (2009) and Rodríguez-Pose and Wilkie (2016) have articulated the important role of host country policy initiatives targeted at foreign-owned firms. The provision of public funding for R&D in foreign-owned subsidiaries presents a crucial conundrum for both academics and policymakers alike. Moncada-Paternò-Castello et al. (2011: 595) argue that a potential negative impact associated with subsidiaries’ R&D projects in a host country, is that “[R&D] [r]esults may be exploited elsewhere”, resulting in “loss of economic benefit [for the host country]”. As highlighted by the European Commission (2017a: 13), a key implication from this feature of multinational firms' globalised R&D strategy is that “policy measures to promote R&D…may only yield a small number of jobs and only provide a weak stimulus to growth when foreign-owned firms decide to produce abroad”. Therefore, there is an important dilemma for host country policymakers. On the one hand, allocating public R&D funding to foreign-owned subsidiaries offers a unique opportunity for host countries to leverage R&D-driven firm performance improvements for the domestic economy. On the other hand, subsidiaries may transfer the knowledge produced by government-funded R&D to other locations, and not commercialise R&D results in the host country. This constitutes a unique risk, that significant amounts of scarce public R&D funding will translate into little, or no firm performance payoffs in the host economy.

The European Commission (2007), when considering R&D internationalisation, has a long tradition of highlighting the de-linking of where foreign-owned subsidiaries conduct R&D, and where they commercialise R&D. They highlight this phenomenon as a major concern for policymakers in host countries, seeking to achieve R&D-driven firm performance improvements in the foreign-owned subsidiaries located in their economies. Subsequent reports by the European Commission (2017a, 2017b) emphasise the continued significance of this issue. These reports specifically highlight the case where public R&D support is allocated to foreign-owned subsidiaries, and does not lead to in situ commercialisation of the publicly-supported R&D results. The in situ commercialisation of publicly-supported R&D in foreign-owned subsidiaries is important for host countries, when seeking to reap the benefits of expanding production activities, as well as garnering the direct economic benefits that flow from such activities (see also Holm et al., 2003; Farah et al., 2021). As the European Commission (2017a, 2017b; 2007) makes clear, in situ commercialisation of R&D represents a distinct and under-researched dimension of how public R&D support for FDI pays off for the host economy. It is this issue which forms the focus of the current paper.

Our study makes two distinct contributions to the literature on public funding for R&D. Firstly, to the best of our knowledge, ours is the first study to examine the above issue based on detailed firm-level panel data. Several studies emphasise the importance of public funding for R&D in attracting and leveraging economic gains from foreign-owned subsidiaries (Agarwal et al., 2007; Feldman & Schipper, 2007; Rodríguez-Pose & Wilkie, 2016). Indeed, Rodríguez-Pose and Wilkie (2016: 2031) recommend that countries should tailor their suite of R&D supports to “increase the appeal of their respective countries to the foreign R&D activities of MNEs”. However, the evidence base underpinning this important policy recommendation is remarkably scant. Previous analyses (e.g. Aerts, 2008; Görg & Strobl, 2007; Liu et al., 2016) have provided some insights. However, the majority of earlier studies have been partial in nature, relying mainly on small, cross-sectional datasets, with limited information on R&D support instruments, and firms’ R&D and performance outcomes. Our study builds on this work by overcoming these limitations, thus providing new insights on the drivers of subsidiary R&D and firm performance in a host country.

Our study's second contribution centres on the differential impacts of R&D grants and R&D tax credits in foreign-owned subsidiaries. In most countries throughout the world, R&D grants and R&D tax credits are the main policy instruments used by governments to provide R&D support to firms (Cunningham & Link, 2021; Lenihan et al., 2020). Previous research has conceptualised key differences in how each type of public R&D support may impact foreign-owned firms’ R&D and performance in a host country (IMF, 2016; European Commission, 2017a). This distinction suggests that R&D tax credits may be particularly powerful at driving subsidiary R&D. Moreover, *the type of R&D* stimulated by R&D grants may actually be more effective at driving subsidiary performance (European Commission, 2017b). Although some recent research has examined both policy instruments in the general population of firms (Marino et al., 2016; Dumont, 2017; Nilsen et al., 2020; Petrin and Radicic, 2023), their relative effectiveness in foreign-owned subsidiaries is completely unaddressed heretofore. Therefore, we advance this field of research by examining which (if any) type of R&D support is most effective at driving subsidiaries’ R&D and economic performance.

A distinct novelty of our study concerns the construction of a unique panel firm level dataset, containing 24,404 firm-year observations for the period 2007–2016^1^ for Ireland. In our sample, and as is standard in the literature, foreign-owned subsidiaries are defined as those who are more than 50% foreign owned/controlled (González & Pazó, 2008; Marino et al., 2016). Since the *First Programme for Economic Expansion* in 1958, the Irish state has adopted a six decade-long industrial development strategy focused on attracting (and later embedding) FDI to stimulate its economy (DJEI, 2014a). As a result of this strategy, the Irish economy is characterised by a duality, between foreign-owned subsidiaries and domestically-owned Irish firms (Bailey & Lenihan, 2015; Cunningham & Golden, 2015). Therefore, Ireland provides the ideal locale to examine whether public R&D funding drives foreign-owned subsidiaries' firm performance in a host country. Our empirical set-up combines detailed survey data, with access to unique administrative data from all of Ireland's main public R&D support agencies. Drawing on this rich panel dataset, we employ a two-stage estimation similar to that employed by Freel et al. (2019) and Beck et al. (2016). Using this approach, we first analyse whether R&D support induces additional firm-level R&D, and second whether this *policy-induced* R&D is effective at driving firm performance. This is particularly important in the specific case of foreign-owned subsidiaries where the risk exists that, even if public R&D support results in additional R&D spending, publicly-funded R&D may not be commercially exploited in the host country.

Our results demonstrate that funding R&D in foreign-owned subsidiaries does indeed pay off for the host economy. This happens in terms of increasing the R&D expenditure of subsidiaries, which is linked to firm performance improvements (e.g. improved turnover, exports, and gross value added). In addition, both types of R&D support instruments produce similar impacts on subsidiaries' R&D and show similar links to performance outcomes. In terms of policy implications, it is important to note that our results refer to a specific country, Ireland. Ireland is a small, open economy, that many foreign-owned firms use as a gateway to the European Union (EU) market (OECD, 2018). As such, one may expect that R&D support to foreign-owned firms in Ireland will produce particularly high spillovers to other locations outside of Ireland, in cases where the foreign-owned firms commercialise the R&D results of their Irish subsidiaries in other EU countries. In contrast to this, we find that Irish government-supported R&D *is* linked to improved firm performance in subsidiaries *in Ireland*. This is crucial in both the Irish context, and beyond. As Cunningham et al. (2020: 85) note, Ireland’s FDI model “has been studied, and emulated by many other small countries around the world”. As such, our study’s findings are relevant to many other small, open economies, and especially those pursuing a strategy of supporting FDI through public R&D funding.

The remainder of the paper is organised as follows. In Sect. 2, we review the literature on R&D grants and R&D tax credits in foreign-owned subsidiaries, and derive our hypotheses. Section 3 outlines our econometric approach and describes our database. Section 4 presents and discusses our results, while Sect. 5 offers a conclusion and discussion of potential implications for policy, along with limitations and possible avenues for future research.

## Theory and hypotheses

The rationales put forward to justify allocating scarce public finances to support foreign-owned subsidiaries’ R&D, are broadly the same as those for firms more generally. The key argument states that firm-level R&D makes a unique and crucial contribution to firm performance, which underpins economic growth and productivity gains (Appelt et al., 2016; Coccia, 2018). However, firms struggle to appropriate sufficient returns from their R&D due to knowledge spillovers (Antonelli, 2020; David et al., 2000). This dis-incentivises R&D investment, and produces a level of private R&D below the social optimum (Hall & Van Reenen, 2000; Smith et al., 2018). The presence of these market failures signals a need for policy intervention (Busom et al., 2014; Sofka et al., 2022). Therefore, policymakers seek to incentivise private R&D, in anticipation that this will contribute to firm performance benefits (Freel et al., 2019; Link & Scott, 2012).

Arrow’s (1962) articulation of the classic market failure rationale for supporting private firms’ R&D with public funding has come to dominate the thinking of most policymakers and funding agencies in this field (Bleda & Del Rio, 2013; OECD, 2020). Under Arrow’s (1962) conceptualisation, the quasi-public good qualities of knowledge, which lead to non-full appropriability and hence private under-investment in R&D, are a weakness. This is because they limit the production of new knowledge, and hence diminish the opportunities for increasing productivity. The commonly held view is that policy can correct for this weakness, using public funding (Antonelli & David, 2016; Antonelli & Link, 2015). However, Antonelli (2020) has hypothesised that the quasi-public good qualities of knowledge are not a weakness, but indeed a key strength of knowledge. Non-full appropriability means that many firms can benefit from ‘the same’ knowledge, which means that knowledge is associated with dynamically increasing returns to investment at the societal level. Based on this, Antonelli (2020) further hypothesises that, as opposed to correcting for market failures, policymakers should allocate public R&D support to increase the flow of private R&D as much as possible. Moreover, Antonelli (2020) argues that firms, sectors, and specific technologies where the additionality and potential knowledge spillovers will be greatest, should be the main target for R&D support (i.e. as opposed to where the market failure is greatest). It is important to note that Arrow’s (1962) argument is still powerful in the case where many firms do not add to the global stock of knowledge, but just profit from using the knowledge others have produced (Dimos & Pugh, 2016; Klette et al., 2000). In this case, not investing in R&D, but rather using the R&D results of others (through knowledge spillovers), can be the more profitable option for a firm, resulting in underinvestment in R&D (Becker, 2015; Vanino et al., 2019). Notwithstanding this caveat, Antonelli (2020) advocates a strong additionality requirement as the basis for allocating public R&D support to firms. Based on Antonelli’s (2020) hypotheses, firms would be required to increase their R&D spending by an amount at least equal to the level of R&D support received. Although public R&D support instruments and programmes examined previously in the literature were not based on Antonelli’s (2020) hypotheses, it is important to consider them in the context of this recent theoretical development. Therefore, this section reviews the literature on public R&D support, and particularly the case of foreign-owned subsidiaries, with Antonelli’s (2020) hypotheses in mind.

### Internationalisation of R&D: Benefits to the host country

In the specific case of foreign-owned subsidiaries, the rationale for public support for firm-level R&D is related to potential payoffs accruing to the host country from the in situ commercialisation of R&D by this subset of firms (Guimón, 2011). Foreign-owned subsidiaries make a significant contribution to national R&D in many host countries. Such subsidiaries have been shown to have a major impact on the national innovation systems of the host country (Belitz & Mölders, 2016; Dachs, 2017; Papanastassiou et al., 2020), and can significantly contribute to the economic development of the host country (Amendolagine et al., 2022; García-Sánchez et al., 2016). Specifically, foreign-owned subsidiaries’ R&D can engender structural change that leads to an increased share of technology-intensive firms, upgrading of domestic firms to higher value-added activities within global value chains, and the creation of technological start-ups (Amendolagine et al., 2022; Dachs, 2017).

Domestic firms can benefit from positive spillovers from the subsidiaries of foreign-owned multinationals through linkage effects (García-Sánchez et al., 2016; Nuruzzaman et al., 2019). Knowledge spillovers arising from various channels, such as cooperation with domestic firms, as well as linkages with local suppliers, customers, universities and research institutions, contribute to upgrading domestic industries and enhancing the innovative capabilities of the host country (Belitz & Mölders, 2016; Vo et al., 2021). The extant literature on FDI spillovers suggests that knowledge spillovers from foreign-owned subsidiaries to domestic firms arise from technology developed in the parent firm (Caves, 1974; Perri & Andersson, 2014). When such technology transfers occur, interactions between the domestic firm and foreign-owned subsidiaries may result in the outflow of local knowledge, which allows the host country firms to exploit the foreign-owned subsidiaries’ technology (Haskel et al., 2007; Perri & Andersson, 2014). Knowledge spillovers also derive from employee mobility from foreign-owned subsidiaries to domestic firms (Cunningham et al., 2020). When previous employees of foreign-owned subsidiaries are hired by domestic firms, the latter may benefit from the knowledge and skills such employees bring with them (Guo et al., 2021; Nuruzzaman et al., 2019; Sofka et al., 2014).

Foreign-owned subsidiary embeddedness is increasingly recognised as a critical factor in generating new knowledge and creating spillovers in the host country (Ghahroudi et al., 2022; Jankowska et al., 2021). To gain access to, and exploit knowledge generated by domestic firms or within the wider multinational group, the foreign-owned subsidiary must be adequately embedded in the host country, known as external embeddedness; and its own global network, known as internal embeddedness (Achcaoucaou et al., 2017; Pu & Soh, 2018). On the one hand, external embeddedness relates to close ties between the foreign-owned subsidiary and actors and organisations (e.g., suppliers, customers, universities) within the host country’s business networks (Davy et al., 2021; Figueiredo, 2011). On the other hand, internal embeddedness describes the links between the foreign-owned subsidiary and its parent’s network (Ferraris et al., 2020); that is, how well integrated the foreign-owned subsidiary is within the parent firm’s network. According to Figueiredo (2011), dual embeddedness (i.e. internal *and* external embeddedness) is a crucial channel, through which foreign-owned subsidiaries can access and share valuable and specific knowledge. It is by combining internal and external sources of knowledge, that firms can generate new ideas which can help stimulate product innovation (Papanastassiou et al., 2020; Vo et al., 2021).

Overall, the internationalisation of R&D offers considerable benefits (such as knowledge and technology spillovers) to the host country (Guo et al., 2021; Jankowska et al., 2021). In two major reports, the European Commission (2017a, 2017b) suggests that public support for firm-level R&D may be necessary, to ensure the host country fully realises these benefits from foreign-owned subsidiaries’ R&D, whilst minimising the associated risks (e.g. increased competition in factor markets). Public R&D support helps host countries to realise the benefits of foreign-owned subsidiaries’ R&D by enhancing the absorptive capacity of domestic firms (Papanastassiou et al., 2020). Moreover, public R&D support helps host countries in minimising the risks associated with foreign-owned subsidiaries’ R&D, by increasing R&D collaboration between domestic firms and foreign-owned subsidiaries (Hottenrott & Lopes-Bento, 2014).

Although the use of scarce public funds to support firm-level R&D in foreign-owned subsidiaries delivers payoffs to the host country (as detailed above), embeddedness has been identified as a critical factor in whether such payoffs are fully realised (Belderbos et al., 2015). However, as detailed by Cunningham et al. (2020) and the European Commission (2017a, 2017b), embeddedness is not the only condition under which host countries benefit from foreign-owned subsidiaries’ R&D. Positive returns for the host country from foreign-owned subsidiaries’ R&D can also occur when the subsidiary does not have strong linkages to the local economy. For example, foreign-owned subsidiaries may expand production activities based on innovations emerging from publicly-funded R&D. The findings of Sofka et al. (2022) suggest that foreign-owned subsidiaries reap greater returns from R&D subsidies in their innovation output than domestic firms, increasing their competitiveness. This in turn can enable foreign-owned subsidiaries to better leverage other host-country advantages, such as high dynamics of the business environment (Holm et al., 2003) or generous corporate taxation rules for foreign-owned subsidiaries (Farah et al., 2021). Moreover, García-Sánchez et al. (2016) find that foreign-owned subsidiaries do not necessarily engage in local host country R&D networks. Rather, their contribution to local networks appears to be economic, instead of technological. The R&D activities of foreign-owned subsidiaries in host countries may also have an enhancing effect on the level and quality of local human capital (Dachs, 2017). For example, if the foreign-owned subsidiaries establish R&D labs which generate additional demand for researchers (Dachs, 2017).

### The impact of public R&D support on firm-level R&D, innovation, and firm performance outcomes

The two main public support instruments, R&D grants and R&D tax credits, influence firms' R&D through different mechanisms (Hall & Van Reenen, 2000). R&D tax credits are deductions from a firm's corporation tax, which lower the marginal cost of R&D, and incentivise firms to invest into new or larger R&D projects (Rao, 2016). R&D grants are usually provided to firms for specific R&D projects that are prioritised by funding agencies (David et al., 2000; Hogan et al., 2021). While R&D tax credits can be claimed after the R&D expenditure has occurred (Elschner et al., 2011), firms usually apply for R&D grants prior to starting an R&D project (Henningsen et al., 2015). R&D grants are often allocated through a competitive process, based on certain selection criteria (Giga et al., 2022; Lanahan, 2016). These criteria are designed to ensure that public money is only used to fund additional R&D investment (i.e. the concept of input additionality), and not substitute for private investment (Hud & Hussinger, 2015). By providing direct support, R&D grants raise the marginal rate of return on R&D, as opposed to reducing the marginal cost (Petrin & Radicic, 2023).

The empirical literature on the impact of public R&D support on different firm-level outcomes is extensive and multifaceted (e.g. Klette et al., 2000; Guellec and Van Pottelsberghe De La Potterie, 2003; Bérubé & Mohnen, 2009; Takalo et al., 2013a; Zúñiga-Vicente et al., 2014; Czarnitzki & Hussinger, 2018; d’Andria et al., 2018). It is an area of research which continues to gain significance and moreover, to witness ongoing investigation (for examples of the most recent evidence see Cerulli et al., 2022; Heijs et al., 2022; Ning et al., 2022; Dai & Chapman, 2022; Lee et al., 2022).

As detailed by Antonelli and David (2016), studies analysing the firm-level effects of public R&D support focus on three distinct processes, which entail different outcome variables: (1) Input additionality, which captures the level of R&D conducted by supported firms; (2) The knowledge production function, which focuses on firm-level innovation; and, (3) The technology production function, which focuses on firm performance. Our study is focused on the impact of policy-induced R&D on firm performance. Therefore, each of these literatures are relevant when identifying what research gaps currently exist, and in specifying the contributions of our analysis. As such, we examine each of them in turn (paying particular attention to points one and three above, as these are most directly relevant to our study).

Firm-level R&D is the most immediate outcome targeted by public R&D support (OECD, 2020). Policymakers seek to compensate firms as their R&D investments are not fully appropriable, due to the quasi-public good nature of knowledge (Antonelli & Link, 2015; Becker, 2015; Huergo et al., 2016). Studies examining the impact of R&D grants and R&D tax credits on firm-level R&D have produced mixed results. Several early studies highlighted the tendency of firms to substitute public funding for their own private R&D investment (Busom, 2000; David et al., 2000; Duguet, 2004). More recent work focused on detecting actual additionality shows that, in many instances, R&D grants and R&D tax credits often crowd out firms’ private R&D spending (Dumont, 2017, 2019; Marino et al., 2016; Takalo et al., 2013b; Thomson, 2017). Indeed, even in the case of selective R&D subsidies targeted at small firms in biotechnology, Choi and Lee (2017) only find evidence for a weak effect on R&D. However, other studies find the opposite result, that is, that R&D tax credits (Dai & Chapman, 2022; Holt et al., 2021; Sterlacchini & Venturini, 2019) and R&D grants (Lee et al., 2022; Mardones & Zapata, 2019; Petelski et al., 2020; Szücs, 2020) drive input additionality. In addition to the theoretical reasons articulated above (i.e. by Antonelli, 2020), Vanino et al. (2019) also highlight that mixed results are likely due to different datasets, methods, and country contexts for each study. In particular, Vanino et al. (2019) draw attention to the fact that most studies only have access to one type of R&D policy instrument, which leaves them unable to account for the likely influence of other R&D supports in their analyses.

In recent years, a small but rapidly expanding literature has developed, which examines the impact of both R&D grants and R&D tax credits on firm-level outcomes (e.g. Busom et al., 2014; Dumont, 2017; Marino et al., 2016; Neicu et al., 2016; Nilsen et al., 2020). This relatively limited set of detailed empirical studies demonstrates the importance of including *both* R&D grants *and* R&D tax credits in the same analysis. Only then will we get a true picture of the contribution of each R&D support type to firm-level R&D and performance. This is because both policy instruments are designed to stimulate input additionality. As such, examining the effectiveness of one (e.g. R&D grants), whilst not including the other (e.g. R&D tax credits), can act as a crucial omitted variable, and obscure any observed effects (Petrin & Radicic, 2023). In addition, given their co-existence, the ability to directly compare relative effects is crucial to our understanding of the effectiveness of each policy instrument individually. For example, an analysis focusing solely on R&D grants may reveal that this policy instrument is effective at stimulating firms’ R&D. However, if R&D tax credits are also included, the observed effectiveness of R&D grants may change. This would thus alter our understanding of the impact of R&D grants on firms’ R&D.

In terms of the innovation process that links firms’ R&D and firm performance, previous studies have generally found a positive and significant effect of public R&D funding on firm-level innovation. For instance, in a sample of firms based in Canada, Bérubé and Mohnen (2009) show that firms which receive both R&D grants and R&D tax credits introduce more radical product innovations, relative to firms that received only R&D tax credits. Neicu et al. (2016) report similar findings for firms in Belgium, as do Mulligan et al. (2019) for a pan-European dataset including firms in Germany, Greece, Ireland, Portugal, and Spain. In addition, Corredoira et al. (2018) highlight that public R&D support plays a key role in driving breakthrough inventions in important technology fields, that non-supported firms tend to avoid. However, Acemoglu et al. (2018) sound a cautionary note, highlighting that R&D subsidies allocated to incumbent firms can trigger barriers to exit, thus supporting the survival and expansion of firms with weak innovative capabilities. Notwithstanding this, the positive effects of public R&D support on firm-level innovation are generally confirmed in the most recent literature (see e.g. Gao et al., 2021; Giga et al., 2022; Ning et al., 2022).

Beyond R&D and innovation impacts, a smaller but still significant literature also exists which examines the relationship between public R&D funding, firm-level innovation, and firm performance. Reviewing this literature, Becker (2019: 13) concludes that “overall, findings confirm the existence of a positive relationship between public R&D support, innovation and firms performance”. This finding is echoed in another review by Mitchell et al. (2020: 122), who note that “[t]here is some evidence of positive effects on employment, productivity, sales and added-value”. However, both reviews highlight the need for future research, with a particular focus on appropriate data and method. Becker (2019: 13) calls for “[g]reater access to, and use of, administrative data”, while Mitchell et al. (2020: 122) state that the results of previous analyses “need to be validated using more robust methods”.

Indeed, although the overall effectiveness of public R&D support at driving firm performance is positive, previous research does present a somewhat mixed picture. Positive effects are found in terms of improving firms’ financial performance (Howell, 2017; Zhao & Ziedonis, 2020), increasing their investments (Seidel & Von Ehrlich, 2015), competitiveness (Aguiar & Gagnepain, 2017), employment (Link & Scott, 2012, 2013; Criscuolo et al., 2019; Lanahan, 2021), investment in tangible and intangible assets (Cerulli et al., 2022; Mulier & Samarin, 2021), value added (Duch et al., 2009; Solomon, 2021), and productivity (Cin et al., 2017). However, other studies do not find a positive and significant link between public R&D support and a wide range of firm-level performance measures (e.g. De Blasio et al., 2015; Karhunen & Huovari, 2015; Wang et al., 2017).

When examining the role of public R&D support in driving firm performance, it is critical to disentangle the impact of publicly-funded R&D, from the impact of firms’ purely privately-funded R&D (i.e. R&D spending that would have happened even in the absence of the support). This is important because, as discussed by Freel et al. (2019), increased R&D and innovation are only intermediate steps *en route* to public R&D support driving firm performance (see also Duch et al., 2009). However, as noted by Solomon (2021: 540) “this mechanism cannot be taken for granted”. Firms may devote their own private R&D to R&D projects which they consider core to their business strategy, and only use public funding for projects with lower expected returns (Czarnitzki & Hussinger, 2018). Alternatively, public R&D support could be used to fund new, additional R&D projects, which the firm considers key, but could not be undertaken in the absence of the support (Czarnitzki & Lopes-Bento, 2013; Szücs, 2020). Solomon’s (2021) analysis finds that public R&D funding is not effective at driving firm performance, when its effects are disentangled from the rest of the firm’s R&D portfolio. However, as noted by Vanino et al. (2019), most studies to date have focused on the direct effect of public R&D support on firm performance. As such, there is no clear consensus on the relative effectiveness of publicly-funded and privately-funded R&D at driving firm performance. Evidence on the relative effectiveness of policy-induced R&D from R&D grants and R&D tax credits, is completely absent in the prevailing literature.

### Impact of public R&D support on foreign-owned subsidiaries

Many studies have suggested that R&D support may have markedly different impacts in foreign-owned subsidiaries compared to domestic firms (Görg & Strobl, 2007; OECD, 2015). To account for these different impacts, most studies treat foreign ownership as a control variable, when examining the impact of R&D supports on firm-level outcomes (e.g. Beck et al., 2016; Cerulli & Potì, 2012; González & Pazó, 2008; Hud & Hussinger, 2015; Marino et al., 2016; Vanino et al., 2019). However, the use of foreign ownership as a control variable alone, provides little insight regarding the impacts of public R&D funding within the specific case of subsidiaries. In this study, we specifically analyse foreign-owned subsidiaries as a distinct group of firms. We examine the impact of public R&D support on this group, by comparing foreign-owned subsidiaries that received public R&D support, relative to similar foreign-owned subsidiaries that did not receive public R&D support.

Treating foreign-owned subsidiaries as a distinct group of firms follows the international business literature, which emphasises the importance of group membership and whether firms are domestically or foreign-owned (see e.g. Kwon & Park, 2018; Cozza et al., 2021; Sofka et al., 2022). As discussed in Sect. 2.1, membership of a wider multinational group offers subsidiaries key advantages over wholly domestic firms, regardless of whether the firm is of foreign or domestic ownership (Cook et al., 2013). Such advantages derive from the subsidiaries’ access to global resources (e.g. superior knowledge and technology), through its links with the parent firm and other subsidiaries within the MNE global network (Un & Cuervo-Cazurra, 2008). However, it is important to note that our analysis does not aim to suggest whether it is better to spend public money on foreign subsidiaries, as opposed to domestic firms (or vice versa). To achieve this, we would need to compare foreign-owned subsidiaries with comparable domestic firms (i.e. to domestic business groups), which is something we cannot do based on our data, as described in Sect. 3.

MNE subsidiaries have several unique features which may result in distinct effects of policy intervention. For instance, while financing R&D is a key issue for most firms, MNE subsidiaries can usually allocate considerable internal funds for R&D (Dachs et al., 2008; Sadowski & Sadowski-Rasters, 2006). Additionally, MNE subsidiaries often enjoy ready access to the parent firm’s internal funds, as well as international capital markets (Dachs & Peters, 2015; Sachwald, 2008). This can help subsidiaries to spread R&D-related risk across a wider variety of projects within the enterprise group, which reduces the risk inherent in R&D (Girma et al., 2003).

MNE subsidiaries' advantages in terms of R&D financing and risk mitigation can also influence decisions about using certain types of public R&D support schemes (Hud & Hussinger, 2015). R&D tax credits reduce the overall cost of R&D, and thus factor into multinational firms’ overarching global R&D strategies (Rao, 2016). In this way, the availability of R&D tax credits can influence MNE subsidiaries’ decision making on where to locate R&D capacity (IMF, 2016; European Commission, 2017b). In terms of direct R&D funding, policymakers may offer R&D grants, designed to incentivise foreign-owned subsidiaries to undertake specific types of R&D projects. For example, in certain thematic areas, or in co-operation with certain partners. R&D grants may also be used to support the establishment of a long-term R&D facility in the host country (Montmartin & Herrera, 2015). This type of direct funding will be particularly important where the multinational parent has yet to decide where to locate specific R&D capacity; and could act as an anchor for a foreign-owned subsidiary to begin, or indeed increase R&D spending in a host country (Appelt et al., 2016). In addition, if the host country offers grant funding for a certain thematic priority (e.g. R&D in a specific scientific field), foreign-owned firms may enjoy ready access to this funding call, by relying on the global R&D network within the wider multinational group (Görg & Strobl, 2007). This may result in the parent firm relocating specific thematic R&D capacities to the host country subsidiary, and away from other locations in the multinational's global network.

In terms of previous empirical findings, a lacuna of literature exists which has focused specifically on the effect of R&D support in foreign-owned firms. Görg and Strobl (2007) found that R&D grants did not produce input additionality in foreign-owned subsidiaries. However, the more common finding is that public funding for R&D has a positive and significant effect on foreign-owned subsidiary R&D. For example, previous studies which find a positive effect of grants on foreign-owned subsidiary R&D include Aerts (2008) for Flanders, Giroud et al. (2012) for South Korea, Rodríguez-Pose and Wilkie (2016) employing European data, and, most recently, Sofka et al. (2022) in a sample of firms based in Germany. In terms of R&D tax credits, both Rao (2016) and Acheson and Malone (2020) find that this form of indirect support drives R&D in foreign-owned firms, in the United States and Ireland respectively. Using more aggregate indicators of public R&D funding in China, Liu et al. (2016) reinforce this overall trend of positive and significant effects on R&D.

Therefore, based on theory and previous empirical findings, we suggest the following hypothesis:

#### Hypothesis 1

R&D grants and R&D tax credits produce input additionality in foreign-owned subsidiaries.

We now turn to likely differential effects of R&D grants and R&D tax credits (Aiello et al., 2019; Hall & Van Reenen, 2000; Petrin & Radicic, 2023). In the general population of firms, decisions about applying for public R&D support and how to use it, are typically part of an in-house strategic decision-making process (Busom et al., 2014), with managers aligning the use of R&D support with the firm's strategic priorities (OECD, 2015). However, in the specific case of foreign-owned subsidiaries, a significant level of decision-making power rests with the parent firm in the home country (Sachwald, 2008; Sofka et al., 2022). For example, the use of R&D tax credits may be part of the multinational firm's overall taxation strategy, with major decisions taken by the financial department in the parent firm's headquarters (Rao, 2016). Direct government funding through grants for specific R&D projects is more likely to contribute to what can be termed 'core projects' (Görg & Strobl, 2007). Thus, it is likely that grant recipients are more prepared to invest their own private funding into these projects, alongside the public R&D funding (Vanino et al., 2019). This may disadvantage foreign-owned subsidiaries, who do not have the final say regarding what to prioritise in their R&D strategy (Guimón, 2009). When decisions on where to locate R&D are taken on a global basis, and decided upon by the parent firm, foreign-owned subsidiaries may not be able to tailor applications for direct R&D funding to the specific requirements of host country funding agencies (Hud & Hussinger, 2015,2015).

In contrast with R&D grants, relatively few studies have examined the impact of R&D tax credits on foreign-owned subsidiaries. However, the limited previous literature that does exist, suggests that this policy instrument tends to be most effective in older, resource-abundant firms, with significant pre-existing R&D capacity (Acheson & Malone, 2020; Busom et al., 2014; Neicu et al., 2016). Additionally, the fact that R&D spending needs to occur before a firm can claim an R&D tax credit, makes it less attractive to resource-constrained firms, and non-R&D active firms (Busom et al., 2014). Due to their multinational group structure, foreign-owned firms can relocate significant R&D spending to countries where the cost of conducting R&D is lower (Montmartin & Herrera, 2015). Therefore, R&D tax credits can lead to large-scale input additionality in foreign-owned subsidiaries, resulting from a relocation of R&D resources within a multinational firm (Appelt et al., 2016). In this regard, the European Commission (2017b) suggest that the impacts of R&D tax credits may outmatch those of R&D grants, in terms of driving subsidiaries’ R&D in the host country. This suggests the following hypothesis:

#### Hypothesis 2

R&D tax credits will produce higher input additionality in foreign-owned subsidiaries, relative to R&D grants.

The distinct features of foreign-owned subsidiaries are also likely to result in performance differences, relative to domestic firms (Dachs & Peters, 2015), and enhance the effectiveness of R&D induced by public support (Giroud et al., 2012). For instance, subsidiaries often possess superior access to assets in terms of knowledge, technologies, brands, and distribution networks (Dachs et al., 2008); as well as organisational and managerial capabilities and practices (Moncada-Paternò-Castello et al., 2011). Due to foreign-owned firms' multinational structures, these superior assets can be transferred across the international group, from the parent firm based in the home country, or from other foreign-based affiliates (Aerts, 2008). Dachs and Peters (2015) highlight that learning from the experiences of other multinational affiliates is an advantage when it comes to conducting R&D, and crucially, using R&D to drive firm performance. For example, foreign-owned firms can bring new products to market more easily, distribute them more widely, and implement process innovations more effectively than other firms (Guimón, 2009). This is because subsidiaries benefit from the experiences of multinational affiliates in other countries, with similar products and technologies (Moncada-Paternò-Castello et al., 2011). These features can help subsidiaries achieve higher performance outcomes, relative to other firms (Sadowski & Sadowski-Rasters, 2006; Vanino et al., 2019). However, it must be noted that the positive impacts of R&D support on subsidiary performance in the host country may be limited. This could occur if the subsidiary’s parent firm transfers R&D results obtained from publicly-supported R&D, to other locations of the corporate group outside of the host country (European Commission, 2017a). Such transfers are more likely, the more a corporate group organises R&D within global networks (Ito et al., 2021; Kwon & Park, 2018). Nevertheless, based on the weight of evidence presented above, it is likely that the distinct features of foreign-owned subsidiaries, will outweigh likely spillovers of R&D results to associate firms outside the host country. As such, we suggest the following hypothesis:

#### Hypothesis 3

Policy-induced R&D from R&D grants and R&D tax credits, is positively linked to firm performance in foreign-owned subsidiaries.

Finally, we move to consider the relative impacts of policy-induced R&D from R&D grants and R&D tax credits on subsidiary performance in the host country. The distinct features of subsidiaries noted earlier, can interact with each policy instrument in somewhat different ways, leading to potentially different impacts on firm performance. As noted in Sect. 2.2, the R&D firms undertake which is supported by tax incentives is not tied to any specific R&D project deliverables (Elschner et al., 2011). This differs significantly from R&D grants, where firms must apply for R&D funding, and indicate what the firm would not be able to achieve without the support (Hud & Hussinger, 2015). As such, it is possible to suggest that the *type of R&D* stimulated by R&D grants in foreign-owned subsidiaries may differ to that from R&D tax credits (European Commission, 2017b), because it is more linked to host country-specific activities (IMF, 2016). Therefore, direct R&D support is likely to be more closely associated with the subsidiary's economic activities within the host country (European Commission, 2017a). In the case of R&D tax credits, a likely increase of R&D in the foreign-owned subsidiary, will often result from a reallocation of R&D capacities within the overarching multinational group, towards locations with lower R&D user cost (Rao, 2016). The output of the subsidiary's additional R&D efforts (i.e. new knowledge or new technologies) will feed into the multinational's global knowledge network, and may be commercialised as part of a global innovation strategy (Moncada-Paternò-Castello et al., 2011). Therefore, it is less likely that the firm performance benefits arising from R&D tax credit-induced additional R&D spending will emerge in the host country, as compared to additional R&D resulting from R&D grants. Based on these factors, we formulate our final hypothesis:

#### Hypothesis 4

Policy-induced R&D from R&D grants will have a stronger link to foreign-owned subsidiaries' performance, relative to R&D tax credits.

## Methodology and data

For our empirical approach, we adopt a two-stage method, similar to Freel et al. (2019) and Beck et al. (2016). Firstly we examine the impact of government R&D support on firm-level R&D, distinguishing between R&D grants and R&D tax credits. For each type of support, we estimate the amount of additional R&D that can be attributed to this support mechanism. Secondly, we estimate the impact of the *policy-induced* R&D from both grants and tax credits, on different measures of firm performance. Before describing our method, we provide necessary details on the policy context of FDI in Ireland, with a particular focus on FDI embeddedness.

### The policy context of FDI in Ireland

We apply the above two-stage method using data on firms based in Ireland. Full details on the policy context of Ireland, as well as detailed descriptions of the R&D grant and R&D tax credit policy instruments used in this study, are provided in Appendix A in the Supplementary material accompanying this paper. To summarise, Ireland has two main enterprise development agencies responsible for awarding R&D grants: (1) Enterprise Ireland (EI), who support domestic Irish-owned firms; and (2) Industrial Development Agency Ireland (IDA), whose sole focus is on attracting and supporting foreign-owned firms. Both EI and IDA implement a number of different R&D grant programmes, which are described in Table A1 in the Supplementary material. In our analysis, all of these programmes are aggregated into one measure per funding agency, capturing all direct R&D grant funding available to firms. In addition, the Irish R&D tax credit provides a 25% refund on qualifying R&D expenditures. The R&D tax credit is available to both domestic and foreign-owned firms undertaking qualifying R&D activity in Ireland. Table A2 in the Supplementary material provides the relative R&D tax credit claims from each firm ownership type.

In terms of policy context, it is important to emphasise the dichotomy in the Irish economy between foreign-owned subsidiaries and domestic Irish-owned firms (Bailey & Lenihan, 2015). As a result of this dichotomy, in the analysis, we split our dataset into two samples: (a) foreign-owned subsidiaries based in Ireland; and (b) domestically-owned Irish firms. In this regard, we note that it is not possible to directly compare the magnitude of our results for domestic and foreign-owned firms, because they come from distinct samples. However, the decision to split our sample is crucial, as it enables us to *verify our results* concerning foreign-owned subsidiaries. For example, should we find that public R&D support has no effect on subsidiaries’ performance, this may suggest that the key risk associated with funding subsidiaries’ R&D has come to pass. However, this may be a spurious conclusion, if the R&D support is simply not effective at driving firm performance *in general*. In this regard, the domestic sample provides a context, where the risk associated with funding subsidiaries is not likely to occur, enabling the verification of our foreign-owned sample findings.^2^ As such, the pattern of results which emerges from the domestic firms’ sample is key to achieving an accurate interpretation of our results for foreign-owned subsidiaries. Our domestic sample consists of 18,920 observations, while our foreign-owned sample contains 5,388 observations. Due to the structure of the Irish economy and the quality of our dataset, our sample of foreign-owned subsidiaries is over twice as large as that used by Girma et al. (2003), four times larger than Görg and Strobl (2007), and over 10 times larger than those used by Acheson and Malone (2020), Liu et al. (2016), Giroud et al. (2012), and Aerts (2008). This is an important feature of our sample, enabling us to overcome many data limitations encountered by previous studies focused on foreign-owned subsidiaries.

Despite the significant strengths of our rich and novel dataset relative to those available to previous studies, we do note one important limitation in relation to our domestic-only sample: We cannot identify whether domestic firms have a group structure. As recently detailed by Cozza et al. (2018) and Sofka et al. (2022), this is a potentially important factor when examining the effects of R&D support in domestic firms. In our analysis, we do not compare domestic firms that were part of a domestic business group, with independent domestic firms.^3^ Given this specific limitation of our dataset, the results from our domestic sample must be interpreted with this in mind. We call on future research to examine domestic firms, taking group status into account (assuming data availability for this variable, which is not currently available in the Irish data). Notwithstanding this, the main focus of our analysis is on the subsidiaries of foreign-owned multinational firms based in Ireland. Given that all of these firms, by definition, have a group structure, the issue noted above does not affect our main analysis.

### Selection into treatment: A two-stage approach

As is common in the literature (e.g. Vanino et al., 2019), we refer to firms that receive R&D support as ‘treated’, and all other firms as ‘untreated’. Selection bias is a key issue when evaluating the impact of public support for R&D on firm performance. This issue arises because firms self-select into the treatment (Cerulli and Poti, 2012), and government agencies select better performing firms for funding, the so-called ‘picking winners’ strategy (Dumont, 2017). Thus, recipient firms may not be representative of the population of R&D active firms. Failure to address this form of selection bias, might result in over-estimating the impact of policy supports for recipient firms (Giga et al., 2022).

Several methods have been developed in the existing literature to address the issue of selection into treatment (for a review, see Cerulli and Poti, 2012). To correct for selection into treatment, we apply a two-stage estimation procedure similar to Beck et al. (2016) and Freel et al. (2019). In stage one, we use Propensity Score Matching (PSM) to estimate the contribution of each R&D policy instrument to firms’ R&D expenditure. As noted in Sect. 3.1 above, in our analysis, we split the sample on the basis of firm ownership. As such, we perform the matching analysis separately, in a sample that only contains foreign-owned subsidiaries based in Ireland, and a sample that only contains domestically-owned firms based in Ireland. The key focus of our analysis is on whether foreign-owned subsidiaries based in a host country commercially exploit the results of publicly-supported R&D in situ in the host country. Conducting this analysis in a sample of foreign-owned subsidiaries only ensures that all firms in this sample share a multinational group structure. This is a crucial methodological point because, as detailed by Dach et al. (2008) and Sofka et al. (2022), group membership affects technology sourcing. By splitting the sample we ensure that, in the matching analysis, foreign-owned subsidiaries belonging to multinational groups are: (1) Only ever compared to foreign-owned subsidiaries belonging to multinational groups; and (2) Never compared to domestically-owned firms. Splitting the sample based on firm ownership has been applied previously in the literature, for example, by Girma et al. (2003), Görg and Strobl (2007), Aerts (2008), Hewitt-Dundas and Roper (2010), Giroud et al. (2012), Rodríguez-Pose and Wilkie (2016), Liu et al. (2016), and Wang et al. (2018). We apply a similar approach in our analysis.

Stage two uses the results from stage one, to estimate the impact of the policy-induced R&D on firm performance (relative to privately financed R&D). In line with Freel et al. (2019), the second stage applies a fixed effects panel model, which controls for significant firm-level heterogeneity, when examining the impact of policy-induced R&D on firm performance.

In stage one, we match treated and untreated firms, based on their propensity to receive the treatment. As noted previously, we perform this matching process separately for foreign-owned firms and domestic firms. We do this, to ensure that we do not at any time match a foreign-owned subsidiary with a domestic firm. We calculate the propensity score using a logit model on the probability to receive a treatment:

1 $$Treatment_{kit} = \beta_{0} + \beta_{1} X_{it - 1} + \varepsilon_{it}$$

The term $Treatment_{kit}$ is a dummy variable indicating whether firm *i* received instrument *k* in time period *t*, $X_{it - 1}$ is a set of independent variables for firm *i* in time period *t-1*. The $\beta s$ in Eq. (1) indicate the model coefficients, and $\varepsilon_{it}$ is the error term. Four separate models are estimated, capturing different treatments for different groups of firms: (a) R&D grants for the foreign-owned sample; (b) R&D tax credits for foreign-owned sample; (c) R&D grants for the domestic sample; and (d) R&D tax credits for the domestic sample. In each case, the potential control group consists of only non-treated firms. In the case of R&D grant recipients, the potential control group includes firms which may also claim R&D tax credits (and vice versa).

In the context of a PSM methodology in stage one, it is important to highlight that both R&D grants and R&D tax credits are allocated on the basis of a two-stage decision process (Dumont, 2017). In the first stage, the firm has to decide whether to apply for an R&D grant, or whether to claim an R&D tax credit. In the case of R&D grants, this decision will mainly depend on the type of R&D activities funded under the grant programme, the application cost, and the expected probability of receiving the grant (Smith et al., 2018). In case of R&D tax credits, the decision will depend on whether the firm's R&D expenditure meet the qualifying conditions, the application cost, and the firm's corporate taxation policy (Elschner et al., 2011). In the case of foreign-owned firms, the latter is usually designed by the parent firm, and optimises corporate financing and taxation across all subsidiaries (Guimón, 2009). Consequently, firms who could in theory qualify for R&D tax credits, may not make claims (Busom et al., 2014; Tassey, 2007). In the second stage, public funding agencies decide about applications for R&D grants or R&D tax credit claims, based on eligibility criteria and—in case of grants—project characteristics and budget restrictions (Henningsen et al., 2015). What is important for PSM, is that for both types of R&D support, some firms select into the instrument, while others do not. Additionally, some firms that applied will receive support, while others do not. As such, in line with Czarnitzki et al. (2011) and Guerzoni and Raiteri (2015), PSM can be used for both R&D grants and R&D tax credits, to examine which recipients would have invested in R&D, in the counterfactual case (where they did not receive the government support).

These models generate a firm’s propensity to receive a treatment. For each treated firm *i*, we assign an untreated firm that shows the lowest difference in the propensity score to firm *i*. In line with the recommendation of Cerulli and Poti (2012), we use the 1:1 nearest neighbour matching method for our main stage one analysis, and 1:3 and Kernel density matching to test if our stage one analysis is robust to changes in matching estimator. To avoid so-called ‘bad matches’, the maximum propensity score distance between treated and matched-untreated firms is set to 0.25 times the standard deviation of the propensity scores (Guerzoni & Raiteri, 2015). In addition, matched untreated firms must operate in the same industrial sector, belong to the same firm size category (i.e. micro, small, medium, and large),^4^ and the R&D expenditure data must refer to the same year *t* in which treated firms have received R&D support. As firms can receive multiple treatments over this time period, we ensure that treated firms are only matched with untreated firms from the same year (i.e. treated in *t* is only ever matched with untreated in *t*). In implementing this procedure, we follow the same approach as employed by Beck et al. (2016) and Freel et al. (2019). They note that exact matching on specific firm characteristics, as well as the propensity score, improves the quality of matching.

Drawing on the results of Eq. (1), we calculate the policy-induced R&D for each R&D policy instrument, for both foreign-owned and domestic firms. In doing so, we capture the counterfactual situation of how much a treated firm would have invested in R&D, if it had not received that treatment. The difference between the observed amount and the counterfactual is the policy-induced R&D. This is calculated as follows:

2 $$\alpha_{k,it} = E\left( {{}_{{}}^{T} RD_{it}^{{}} |F_{it} = 1} \right) - E\left( {{}_{{}}^{C} RD_{it}^{{}} |F_{it} = 1} \right)$$

In Eq. (2), $\alpha_{k,it}$ is the treatment effect, which we classify as the policy-induced R&D. We calculate this value for R&D grants as $\alpha_{R\& Dgrant,it}$ and for R&D tax credits as $\alpha_{R\& Dtaxcredit,it}$. Given both values, it is possible to generate a further counterfactual of privately-funded R&D expenditure for treated firms, as follows:

3 $$PrivateRD_{it}^{C} = PrivateRD_{it}^{{}} - \alpha_{R\& Dtaxcredit,it} - \alpha_{R\& Dgrant,it}$$

For untreated firms $PrivateRD_{it}^{C} = PrivateRD_{it}^{{}}$ as $\alpha_{R\& Dtaxcredit,it}$ and $\alpha_{R\& Dgrant,it}$ are equal to zero. Note that $PrivateRD_{it}^{C}$ can be negative, if matched untreated firms show a higher R&D output than treated firms (i.e. crowding-out).

In stage two of our analysis, we examine whether policy-induced R&D ($\alpha_{R\& Dtaxcredit,it}$ and $\alpha_{R\& Dgrant,it}$) is linked to firm performance, by estimating Eq. (4):

4 $$\begin{aligned} FirmPerformance_{it} =\, & \beta_{0} + \beta_{1} \alpha_{R\& Dtaxcredit,it - 1} + \beta_{2} \alpha_{RDgrant,it - 1} \\ & + \beta_{3} PrivateRD_{it - 1}^{C} + \beta_{4} z_{it - 1} + \mu_{i} + \mu_{t} + \varepsilon_{it} \\ \end{aligned}$$

In Eq. (4), $FirmPerformance_{it}$ is measured as, respectively, the natural logarithm of the following variables: Turnover, Gross Value Added (GVA), and Exports. We estimate three separate models in the foreign-owned and domestic samples, with each of these firm performance outcomes as a dependent variable. The term $z_{it - 1}$ is a matrix of control variables for firm *i* in time period *t-1*, and $\beta_{4}$ represents the associated coefficients, while $\mu_{i}$ are time invariant firm fixed effects, and $\mu_{t}$ are time fixed effects. While the year and firm fixed effects control for significant unobserved heterogeneity, the term $z_{it - 1}$ also captures a series of time-varying independent variables (defined in Table 1, alongside all variables used in the analysis). These variables differ to those used in Eq. (1) which examine selection into treatment, because they are more closely associated with firm performance.^5^ In terms of the time lag used in our model between receiving R&D support and any potential firm performance effects, we measure the impact of receiving a treatment in *t-1* on firm-level R&D in *t* (i.e. the current year); we then measure the impact of policy-induced R&D in *t*, on firm performance in *t* + *1*.

**Table 1:** Definition of variables used in the analysis

Variable	Definition
*Stage one dependent variable*	
R&D	Natural logarithm of firm's total R&D expenditure
*Stage two dependent variables*	
Turnover	Natural logarithm of firm's turnover
Exports	Natural logarithm of firm's exports
Gross Value Added (GVA)	Natural logarithm of firm's GVA
*Treatment variables*	
R&D tax credit	Binary variable equal to 1 if a firm claimed an R&D tax credit; 0 otherwise
R&D grant	Binary variable equal to 1 if a firm received direct funding for R&D from Industrial Development Agency (IDA) Ireland or Enterprise Ireland; 0 otherwise
*Control variables*	
Past turnover	Categorical variables capturing the quartile of firms' turnover (natural logarithm): 1 = Firms in the lowest turnover quartile in a given year; 2 = Firms in the second lowest turnover quartile in a given year; 3 = Firms in the second highest turnover quartile in a given year; 4 = Firms in the highest turnover quartile in a given year
R&D above median	Binary variable equal to one if firms' past R&D was above the median R&D expenditure; 0 otherwise
Past public funding for R&D	Binary variable equal to 1 if a firm received an R&D grant or R&D tax credit in the previous period; 0 otherwise
Other R&D support	Binary variable equal to 1 if a firm received any other form of R&D support; 0 otherwise
Firm size	Categorical variables: 0 = Micro if a firm has less than 10 employees; 1 = Small if a firm has 10 or more employees and less than 50 employees; 2 = Medium if a firm has 50 or more employees and less than 250 employees; 3 = Large if a firm has 250 or more employees
Material costs	Natural log of materials and service costs
Unit labour costs	Firm's payroll divided by GVA
Training	Natural logarithm of the firm’s total expenditure on all structured training
Regional R&D	Natural logarithm of the sum of all firms' expenditure on R&D in the region where a firm is based
Sector	Categorical variables representing 12 NACE sectors (defined in Appendix Table C1 in Supplementary material)
Year	Categorical variables: 0 = 2007; 1 = 2008; 2 = 2009; 3 = 2010; 4 = 2011; 5 = 2012; 6 = 2013; 7 = 2014; 8 = 2015; 9 = 2016

NACE is the acronym for ‘nomenclature statistique des activités économiques dans la Communauté européenne’, and is the statistical classification of economic activities used by Eurostat. Other R&D support captures any R&D support which does not provide direct funding through R&D grants or indirect funding through R&D tax credits. The R&D supports in this category are as follows: Innovation Partnerships, Innovation Vouchers, and Science Foundation Ireland research centres. The 26 counties of the Republic of Ireland constitute the regional unit for the Regional R&D variable. For the Regional R&D variable, we aggregate total R&D from our survey to the regional level

Given the panel nature of the data, we estimate Eq. (4) using a fixed effects within group estimator. A significant advantage of the fixed effects estimation procedure, is that it allows for each individual firm to have some special characteristics of its own which are unobserved (Asteriou & Hall, 2011; Wooldridge, 2020). According to the classic econometric text of Gujarati (2003), the fixed effects methodology essentially captures all effects that are specific to a particular firm, which do not vary over time. As Wooldridge (2020) notes, fixed effects within group estimation achieves this by time de-meaning the data for each firm, resulting in the unobserved firm-specific effects being removed. Specific to our analysis, the time invariant factors which may be captured by fixed effects could relate to characteristics of the foreign-owned subsidiaries’ parent and the firm's knowledge base.

In the case of our analysis, previous studies (e.g. Hille & Möbius, 2019; Mairesse & Mohnen, 2010; Véganzonès-Varoudakis & Plane, 2019), suggest that two potential sources of endogeneity may affect the second stage of our estimation procedure: (1) Simultaneity; and, (2) Omitted variables. Beginning with potential endogeneity relating to simultaneity, this may occur where firms’ performance (*y*) impacts their R&D expenditure (*x*), and vice versa, contemporaneously. Hill et al. (2021) detail that if, in addition to *y* causing *x*, *x* causes *y*, both *y* and the error term (*u*) will correlate with *x*. To account for simultaneity, we implement the approach of Holl (2021) and Bartoloni and Baussola (2018), and introduce a lag of all control variables in our model (including our R&D expenditure variables). This is a common approach adopted within the innovation literature to remedy for endogeneity due to simultaneity (Bartoloni & Baussola, 2018; Holl, 2021). The logic underpinning this, is that by introducing a temporal lag in *x*, this variable becomes predetermined, as present values of *y* cannot cause past values of *x* (Bartoloni & Baussola, 2018). This is consistent with much research based on the Community Innovation Survey [see for example Frenz and Ietto-Gillies (2009), and a more recent brief discussion in Aldieri et al. (2021)].^6^

We next turn to the second potential source of endogeneity in our stage two analysis, omitted variables. Endogeneity due to an omitted variable (*q*) occurs when this factor affects *y*, so that when *q* is not modelled, it is included in the residual *u*, and if the omitted variable *q* is correlated with *x*, then *u* is also correlated with *x* (Hill et al., 2021). The potential impact of this omitted variable problem is mitigated through our choice of estimation procedure. The fixed effects within group estimator discussed above explicitly accounts for all time-invariant firm-specific characteristics. In doing so, it reduces the possibility of omitted variables, hence, reducing the potential for the error term to be correlated with *x* due to an omitted variable (Hille & Möbius, 2019).

We perform a number of robustness checks concerning the estimation of Eq. (4). Firstly, as noted above, our main stage two model is estimated using the stage one results from a 1:1 PSM model (i.e. each treated firm is matched with one untreated nearest neighbour). Following Guerzoni and Raiteri (2015), to test the sensitivity of our results to changes in PSM model, we also estimate our stage one models using 1:3 and Kernel density matching approaches. We then use the results from these alternative PSM models in stage two. This enables us to test the robustness of our stage two findings to changes in the way our key policy-induced R&D variables were generated.

Secondly, the issue of ‘policy instrument mix’ has become increasingly important when examining the impact of R&D grants and R&D tax credits on firm-level outcomes [for a recent discussion, see Petrin and Radicic (2023)]. A policy instrument mix is typically defined as firms receiving both an R&D grant and an R&D tax credit at the same point in time (Guerzoni & Raiteri, 2015; Marino et al., 2016; Dumont, 2017; Petrin and Radicic, 2023). Therefore, we perform further robustness tests examining the sensitivity of our stage two analysis to firms receiving each policy instrument individually, or a mix of both instruments. We do this by examining four separate policy-induced R&D variables: 1) Policy-induced R&D from an R&D grant, where the firm received an R&D grant only (no policy instrument mix); 2) Policy-induced R&D from an R&D grant, where the firm also received an R&D tax credit (policy instrument mix); 3) Policy-induced R&D from an R&D tax credit, where the firm received an R&D tax credit only (no policy instrument mix); and 4) Policy-induced R&D from an R&D tax credit, where the firm also received an R&D grant (policy instrument mix).

In addition to the steps undertaken in our main models to account for potential endogeneity, we perform a further robustness test by estimating Eq. (4) using the Lewbel (2012) estimation procedure (see also Lewbel, 2018). A significant advantage of the Lewbel (2012) procedure, is that it uses heteroscedasticity to address the problem of an endogenous regressor, when no external instruments or other such information is available. It is extremely common within innovation surveys for there to be no exogenous or environmental variables which can be used as relevant and valid instruments (for a discussion, see e.g. Mairesse & Mohnen, 2010; and more recently Petrin and Radicic, 2023), as is the case in our analysis. The Lewbel method enables us to overcome this limitation, providing a robustness test of our main results against endogeneity. We perform a series of alternative estimations of Eq. (4) using the Lewbel (2012) method. In each instance, we treat our R&D expenditure variables as endogenous in our estimation procedures, addressing potential endogeneity using heteroscedasticity-based instrumental variables. The Lewbel (2012) procedure has been used extensively in innovation studies as a robustness check to account for endogeneity (see e.g. Naveed & Wang, 2022; Kourouklis, 2021; Czarnitzki et al., 2020; Heim et al., 2017; Liu et al., 2016).

Finally, we note that Eq. (4) evaluates whether policy-induced R&D expenditure and firm performance are positively linked. However, it does not establish a causal relationship in the strict sense. As discussed by De Blasio et al. (2015) and Lanahan (2016), the inability to infer causality when using most available datasets and commonly used methods, is a limitation of current empirical research in innovation studies. However, it is crucial to highlight this issue, to ensure that our findings are interpreted with the above in mind. Therefore, while we can observe whether the policy-induced R&D from R&D grants and tax credits is positively linked to firm performance improvements, we cannot infer a direct causal relationship.

### Data

Our empirical analysis is based on a merged dataset, comprising the Irish Annual Business Survey of Economic Impact (ABSEI), as well as administrative data on R&D grants and R&D tax credits awarded to firms during the period 2007–2016.^7^ ABSEI is an unbalanced annual panel dataset, collected via a postal survey conducted by Ireland's Department of Enterprise, Trade and Employment (DETE), covering a population of approximately 4,000 firms annually, with a response rate of approximately 65% each year. The ABSEI dataset is unique because its sample frame covers all firms that have ever been assisted in any way by Ireland's enterprise development agencies (further details below). As such, ABSEI is specifically designed to cover a large, representative sample of the foreign-owned and domestic firms who receive policy support each year.^8^

The key administrative data comes from Ireland's two main enterprise development funding agencies that provide R&D grants to firms: (1) EI (supports domestic Irish-owned firms); and (2) IDA (supports foreign-owned firms). In concurrence with previous literature (González & Pazó, 2008; Marino et al., 2016), foreign ownership is defined by IDA as whether the firm is more than 50% foreign owned/controlled. Our analysis also draws on novel administrative data from the Irish Revenue Commissioners on firms' R&D tax credit claims. We aggregate all direct grant support from EI and IDA into one variable, capturing whether firms received any R&D grant support in a particular year.^9^ Our merged sample captures 54.26% of the IDA administrative data, 63.74% of the EI administrative data, and 54.04% of the Irish Revenue Commissioners R&D tax credit data. An analysis on the representativeness of our sample, relative to the full populations in these administrative datasets, is presented in Appendix Tables E1, E2 and E3 in the Supplementary material accompanying this paper. These tables demonstrate that our sample is highly representative across a range of different firm-level characteristics.

### Description of variables

Our analysis measures firm-level R&D as the natural logarithm of firms' total R&D expenditure. Although this definition of firms' R&D is common in the literature (e.g. Aiello et al., 2019), some previous studies used firms' R&D divided by turnover (i.e. the ratio) as a dependent variable. However, Alessandri and Pattit (2014) note that the use of such ratio variables can introduce biased correlations into econometric models, which confound the interpretation of results and may lead to spurious findings. Therefore, we follow Alessandri and Pattit's (2014) recommendation to use the logarithm of firms' R&D as the dependent variable, and firms' turnover as a control variable in our stage one analysis. As noted above, our stage two outcome variables are turnover, exports, and GVA. We follow Vanino et al. (2019) and Nilsen et al. (2020) in defining our firm performance outcome variables in logarithms, to help ensure that we avoid heteroscedasticity in our stage two fixed effects models.

For the treatment variables in stage one, we employ binary variables which take a value of one if a firm received an R&D grant or claimed an R&D tax credit in year *t* (or a value of 0 otherwise), over the time period 2007–2016. For further details on the definitions of these variables, see Appendix C in the Supplementary material accompanying this paper. As noted above in Sect. 3.2, we also include several control variables to account for firm-level characteristics. In line with Nilsen et al. (2020), we include a binary variable which captures whether firms were above, or below the sample median R&D expenditure amount in the year before they received any R&D support. As noted by Henningsen et al. (2015), including lagged R&D variables improves the quality of our analysis, by ensuring that past engagement in R&D, for both the control and treatment group, is captured in the matching procedure. In addition, we include four variables capturing the four quartiles of the turnover distribution in our sample, to capture pre-treatment business quality (Vanino et al., 2019). Based on the studies of Busom et al. (2014) and Neicu et al. (2016), we include two dummy variables which capture whether firms received an R&D grant and/or R&D tax credit in the past, and whether firms received any other form of public R&D support^10^ (i.e. non-R&D grant/tax credit support). In addition, we use a series of binary variables which indicate whether a firm is in the micro, small, medium, or large size categories, as well as which sector a firm belongs to (defined in Table 1, and Appendix Table C1 in the Supplementary material accompanying this paper^11^).

Our dataset is large and is well-suited for this analysis. However, as in other studies using survey data [for a discussion see e.g. Colombo et al. (2013) and Mulligan et al. (2022)], ABSEI does not capture some potentially important firm-level information. For example, we would ideally like to include variables capturing skill levels of employees and appropriation mechanisms such as patents, when calculating firms’ propensity to receive public R&D support. Unfortunately, such information is not available in ABSEI, and cannot be merged in from any other data source. Omitting these variables is a limitation of our empirical analysis. However, as discussed by Colombo et al. (2013), all survey datasets have limitations. The variables we do include in our analysis, as discussed above, are all key factors in determining firm-level R&D, and many other factors are captured using fixed effects.

For the stage two model on firm performance, we control for firms' material inputs, unit labour costs, expenditure on training, and the total R&D expenditure in the region the firm is located in (see Table 1). Freel et al. (2019) deem the first two of these variables to be key indicators of price and quality advantages when examining the impact of public R&D support on firm performance. In addition, firms’ knowledge bases and capabilities are critical for growth (Grillitsch et al., 2019; Zouaghi et al., 2018). Indeed, as Belitski et al. (2020) emphasise, firms wishing to compete globally, need to invest in their own knowledge bases. We employ two variables to specifically capture knowledge base effects. On-the-job training has been identified as a critical internal knowledge source, in terms of providing concrete know-how, craft, and the practical skills required in the knowledge production processes (Asheim & Coenen, 2005; Belitski et al., 2020; Dostie, 2018; Grillitsch et al., 2019; Thornhill, 2006; Zouaghi et al., 2018). Expenditure on training is commonly used to capture additions to the knowledge base from on-the-job training (Thornhill, 2006; Diaz-Fernandez, 2017; Grillitsch et al., 2019). Specifically, we use the natural log of training expenditure by the firm, which is consistent with Belitski et al. (2020) and Cozzarin and Percival (2021).^12^ In terms of external knowledge sourcing, studies including Asheim and Coenen (2005), Tödtling and Trippl (2005) and Tojeiro-Rivero and Moreno (2019), highlight the importance of regional context as a critical component of a firm’s knowledge bases, emphasising the importance of regional R&D expenditure. Moreover, Ascani et al. (2020) and Fitjar and Rodríguez-Pose (2015) highlight the importance of local knowledge endowments for a firm’s knowledge base. We capture these factors in our analysis using total R&D expenditure in the region the firm is located in.

Firm-specific determinants of performance such as the accumulated stock of knowledge, market access, reputation, and so forth, are captured by estimating a firm fixed-effects model. In constructing our final sample, we follow a similar procedure to Czarnitzki and Thorwarth (2012), and restrict our sample to only firms that were R&D-active in at least one year over the period 2007–2016. In addition, our sample is characterised by a small number of outliers which have turnover of an order of magnitude above the next largest firm. Therefore, following the recommendation by Falck et al. (2021), we remove the top 1% of firms in this variable. Appendix Table C2 in the Supplementary material accompanying this paper provides summary statistics for all variables used in our sample.

## Empirical results

This section presents and discusses the results of our main analysis. Here we first evaluate the impact of R&D grants and R&D tax credits on foreign-owned subsidiaries' R&D. We then examine the link between policy-induced R&D and firm performance. In addition, we perform a selection of robustness tests for our main analysis.

### Impact of public R&D support on R&D in foreign-owned subsidiaries

To perform our stage one PSM analysis, we match treated and untreated firms based on their propensity to receive the treatment. Table 2 reports the marginal effects from the logit models used to estimate firms' propensity scores (Appendix Table F1 in the Supplementary material accompanying this paper reports the coefficients that these marginal effects are based on). As these results are a necessary first step in our analysis, but are not central to our hypotheses, we present a further discussion of this propensity score estimation in Appendix F in the Supplementary material accompanying this paper.^13^

**Table 2:** Marginal effects from logit model for firms' probability of receiving public R&D funding

	Foreign-owned firms		Domestic firms	
Variables	R&D grants	R&D tax credit	R&D grants	R&D tax credit
R&D above median	0.0765*** (0.00984)	0.0731*** (0.0133)	0.0303*** (0.00550)	0.198*** (0.00694)
Past turnover quartile two	− 0.0478** (0.0194)	0.0671*** (0.0201)	− 0.0187** (0.00841)	0.00766 (0.00908)
Past turnover quartile three	− 0.0441** (0.0206)	0.0593*** (0.0212)	− 0.0181** (0.00912)	− 0.0149 (0.00977)
Past turnover quartile four	− 0.0210 (0.0221)	0.0553** (0.0232)	− 0.0252** (0.0109)	− 0.0203* (0.0118)
Previous public R&D funding	0.252*** (0.0185)	0.358*** (0.0104)	0.224*** (0.00709)	0.250*** (0.00570)
Other R&D support	0.0266** (0.0111)	− 0.0243 (0.0167)	0.0793*** (0.00570)	0.0592*** (0.00700)
Firm size: small	0.0880* (0.0532)	0.143*** (0.0516)	0.00890 (0.00781)	0.0167* (0.00869)
Firm size: medium	0.121** (0.0536)	0.131** (0.0525)	0.00171 (0.0112)	0.0148 (0.0123)
Firm size: large	0.135** (0.0545)	0.0727 (0.0544)	0.00952 (0.0181)	− 0.0419** (0.0200)
Observations	4,051	4,023	15,756	15,756

*p < 0.1; **p < 0.05; ***p < 0.01. Standard errors in parentheses. All coefficients from the logit models are presented as marginal effects, for ease of interpretation. Dummy variables for year and NACE sector are included in the propensity score estimation, but the output is not displayed here. The base category for firm size is micro; the base category for R&D intensity quartile is zero R&D; the base category for turnover is the lowest turnover quartile

To summarise, our results show that some common factors influence both foreign-owned and domestic firms' likelihood to select into using R&D grants and R&D tax credits, such as pre-treatment R&D and previous public R&D funding. However, notable differences also exist. For example, pre-treatment turnover plays a key role in determining whether subsidiaries receive R&D tax credits, but not R&D grants; and has a negative impact on domestic firms' likelihood to receive R&D grants. Overall, these results suggest there are different factors at play when foreign-owned and domestic firms select into using R&D grants and R&D tax credits, supporting our decision to split the sample. Appendix Tables F2 and F3 in the Supplementary material accompanying this paper, demonstrate that our treated and matched untreated samples satisfy the standard balancing criteria tests across all covariates.

Drawing on this matching procedure, Table 3 presents the results from our PSM analysis. We first examine Hypothesis 1, which states that R&D grants and R&D tax credits produce input additionality in foreign-owned subsidiaries. The findings presented in Table 3 demonstrate that both support types have a positive and significant impact on subsidiaries' R&D. Foreign-owned firms that use R&D tax credits spend, on average, €0.803 million per year on R&D, compared to €0.126 million for matched untreated foreign-owned firms, suggesting a treatment effect of €0.677 million. In the case of R&D grants, the treatment effect is higher at €1.005 million. As such, we find strong support for Hypothesis 1. Appendix Table G1 in the Supplementary material accompanying this paper shows that the sign, significance, and magnitude of all estimated stage one results, are robust to changes in the matching estimators.

**Table 3:** Propensity score matching results for the impact of R&D supports on firm-level R&D

Treatment	Treated	Matched untreated	Difference (α)
*Foreign-owned firms*			
R&D tax credit	0.803	0.126	0.677***
R&D grant	1.818	0.813	1.005*
*Domestic firms*			
R&D tax credit	0.194	0.056	0.138***
R&D grant	0.076	0.033	0.043***

*p < 0.1; **p < 0.05; ***p < 0.01. For ease of interpretation, the results in Table 3 are expressed in millions of Euros. Estimation results based on 1:1 nearest neighbour matching. The α term relates to the average policy-induced R&D from each policy instrument

The results are consistent with those reported for direct R&D funding by Aerts (2008), Giroud et al. (2012), Rodríguez-Pose and Wilkie (2016), and Sofka et al. (2022), and for R&D tax credits by Rao (2016) and Acheson and Malone (2020). However, unlike the above studies, we capture both R&D support types in the same analysis. As such, in line with Dumont (2017) and Marino et al. (2016), our findings provide a more precise picture than was possible in previous analyses. This is because our input additionality estimates for each type of R&D support, consider likely effects of the other support mechanism. In addition, we observe a similar pattern of positive and statistically significant results in the domestic firm sample. Taken together, these results reveal that public R&D funding is effective *in general* at driving firms’ R&D. This suggests that we should expect to see follow-on impacts on subsidiaries’ firm performance in stage two, if two conditions are met: (1) the policy-induced R&D from R&D grants and R&D tax credits is used effectively; and (2) foreign-owned subsidiaries commercially exploit the policy-induced R&D in the host country, at least to an extent sufficient to drive a positive and significant impact on firm performance.^14^

We next turn to Hypothesis 2, which states that R&D tax credits will produce larger input additionality in foreign-owned subsidiaries' R&D, relative to R&D grants. In terms of differential effects, it is clear from Table 3 that the magnitude of the result for R&D tax credits is smaller than that for R&D grants. However, the input additionality found for R&D tax credits is statistically much more robust (*p* < 0.01) than for R&D grants (*p* < 0.1). This suggests that R&D tax credits incentivise additional R&D expenditures much more reliably across the entire group of foreign-owned firms, than is the case with R&D grants. The additionality of R&D grants is much more heterogeneous across foreign-owned subsidiaries. Some subsidiaries increase R&D expenditure at a very high rate, resulting in a larger, though less robust average effect.

To examine the statistical significance of the difference in input additionality between the two types of support, we perform a mean comparison test. This is possible because both results come from the same sample of firms (i.e. foreign-owned). Results from this test reveal that there is no statistically significant difference between the impact of R&D grants and R&D tax credits on foreign-owned subsidiaries' R&D. Therefore, we can say that both R&D support types produce a statistically identical percentage increase in subsidiaries' R&D, and thus formally reject Hypothesis 2. These results contrast with Marino et al. (2016), who report generally non-significant, or negative impacts from R&D grants and R&D tax credits on firms' R&D. However, our results are more in line with Neicu et al. (2016) and Dumont (2017), who find that both R&D support types can drive additional firm-level R&D. Our analysis builds on these studies, by confirming their findings in the specific case of foreign-owned subsidiaries operating in a host country, a key sub-group within the general population of firms (Moncada-Paternò-Castello et al., 2011).

As detailed in Sect. 2.2, Antonelli (2020) has highlighted that most (if not all) public R&D support is allocated on the basis of correcting for market failures, arising from the quasi-public good qualities of knowledge (see also Antonelli & David, 2016). As such, public R&D support is designed to restore equilibrium conditions, and bring the production of knowledge back to its societal optimal level (i.e. in line with the work of Arrow, 1962). This provides a basis for understanding why many previous empirical studies report that public R&D support leads to non-full crowding-out, as opposed to actual input additionality (Dimos & Pugh, 2016). However, Antonelli (2020: 649) suggests that, in future, public R&D support should be allocated using a “strong additionality requirement”, where “recipients should increase the levels of R&D performed by an amount equal or larger than the public fund”. The R&D supports we examine in our study were allocated on the basis of correcting for market failures (see e.g. DJEI, 2014b; Irish Comptroller and Auditor General, 2016; Acheson & Malone, 2020; Hogan et al., 2021). Nevertheless, it is informative to analyse whether Antonelli’s (2020) additionality criteria are achieved in our sample, as this represents an important yardstick for assessing the effectiveness of public R&D support.

Therefore, Table 4 presents the ratio of average public R&D support through R&D grants and R&D tax credits, to the average level of input additionality achieved through each support type. Table 4 demonstrates that, on average, €1 of R&D tax credit support leads to €0.68 of additional R&D spending in foreign-owned subsidiaries, and €1.13 in domestic firms. These results suggest that, to some extent, foreign-owned subsidiaries substitute R&D tax credit support for their own private R&D spending. In contrast, R&D tax credits drive true input additionality, of the sort detailed by Antonelli (2020), in domestic firms. In terms of R&D grants, the opposite result holds. Table 4 suggests that €1 of R&D grant support drives €1.22 additional R&D spending in foreign-owned subsidiaries, and €0.42 in domestic firms. This latter result is particularly interesting in light of Antonelli (2020), as domestic firms in Ireland are typically financially-constrained SMEs (DJEI, 2014b), where the classic market failure rationale is most acute. As such, this result of partial crowding-out, but not full crowding-out, is perhaps unsurprising (Dimos & Pugh, 2016). However, our finding that R&D grants drive > €1 of additional R&D spending in foreign-owned subsidiaries is notable. This suggests that direct and targeted R&D support is highly effective in foreign-owned subsidiaries, even under Antonelli’s (2020) strong additionality criteria.

**Table 4:** Average input additionality of R&D grants and R&D tax credits

	R&D tax credit	R&D grant
Foreign-owned subsidiaries	0.68	1.22
Domestic firms	1.13	0.42

This table examines whether, on average, €1 in public R&D support leads firms to invest > €1 or < €1 of additional R&D spending. Figures below one represent firms that invested < €1. Figures above one represent firms that invested > €1. We note that in the case of R&D tax credits, these are provided in bands rather than exact amounts, and that the mid-point of the band is used for this calculation

### Role of public R&D support for firm performance outcomes

Table 5 presents the results from our stage two analysis. We first focus on Hypothesis 3, which states that policy-induced R&D from R&D grants and R&D tax credits, is positively linked to firm performance in foreign-owned subsidiaries. As noted in Sect. 3.2, policy-induced R&D can be negative, if matched non-treated firms have greater R&D than treated firms (i.e. crowding-out). We define our R&D outcome variable in stage one as the natural logarithm of firms' total R&D. As logarithms cannot take a negative value, we convert this figure into actual monetary terms, by calculating the exponential. The monetary amount can have a negative value, which ensures that our stage two estimations include any potential crowding-out effects. Crowding-out occurs in instances where firms substitute some or all of the public R&D support, for their own private R&D spending (David et al., 2000). This often results in lower R&D investment than would have been the case in the absence of the public support (González & Pazó, 2008), and is thus vital to capture in our two-stage analysis.^15^

**Table 5:** The link between policy-induced R&D and firm performance, in foreign-owned subsidiaries and domestic firms

Variable	Foreign-owned subsidiaries			Domestic firms		
	Turnover (log)	GVA (log)	Exports (log)	Turnover (log)	GVA (log)	Exports (log)
Privately-funded R&D	0.00806** (0.00322)	0.00751*** (0.00259)	0.00734** (0.00335)	0.152*** (0.0349)	0.129*** (0.0346)	0.159*** (0.0388)
Policy-induced: R&D tax credit	0.00668** (0.00315)	0.00599** (0.00323)	0.00607* (0.00322)	0.159*** (0.0306)	0.136*** (0.0312)	0.132*** (0.0361)
Policy-induced: R&D grant	0.00775** (0.00337)	0.00618** (0.00293)	0.00703** (0.00352)	0.146*** (0.0355)	0.119*** (0.0352)	0.172*** (0.0397)
Firm size: small	0.113 (0.188)	− 0.0186 (0.170)	0.145 (0.190)	0.381*** (0.0256)	0.412*** (0.0297)	0.346*** (0.0439)
Firm size: medium	0.564*** (0.199)	0.343* (0.185)	0.500** (0.199)	0.751*** (0.0360)	0.775*** (0.0426)	0.663*** (0.0640)
Firm size: large	0.937*** (0.208)	0.671*** (0.199)	0.878*** (0.217)	1.128*** (0.0752)	1.184*** (0.0889)	0.893*** (0.143)
Materials	0.198 (0.169)	− 0.912*** (0.182)	0.145 (0.158)	0.0252 (0.0409)	− 0.787*** (0.0787)	0.0579*** (0.0224)
Unit labour costs	− 0.0454*** (0.0133)	− 0.0534*** (0.0166)	− 0.0540*** (0.0168)	− 0.000532 (0.000561)	0.00211 (0.00175)	− 0.000351 (0.000979)
Training	0.0697*** (0.0174)	0.0831*** (0.0201)	0.0582*** (0.0181)	0.0517*** (0.00585)	0.0541*** (0.00736)	0.0589*** (0.0102)
Regional R&D	0.0136 (0.0418)	0.06244 (0.0538)	0.02296 (0.04668)	0.01592 (0.01937)	− 0.0013 (0.0261)	− 0.0210 (0.0406)
Constant	16.29*** (0.8034)	15.083*** (0.9937)	15.749*** (0.8720)	14.122*** (0.3575)	14.0183*** (0.48204)	13.3334*** (0.7455)
Firm fixed effects	Yes	Yes	Yes	Yes	Yes	Yes
Year fixed effects	Yes	Yes	Yes	Yes	Yes	Yes
Observations	3,600	3,548	3,439	14,168	13,864	12,387
R^2^	0.235	0.191	0.199	0.238	0.181	0.167

*p < 0.1; **p < 0.05; ***p < 0.01. Robust standard errors in parentheses. The policy-induced R&D variables correspond to the α term defined in Eq. (2). The number of observations changes in each model due to missing values in the dependent variables. For ease of interpretation, the variables ‘Privately-funded R&D’, ‘Policy-induced: R&D tax credit (α)’, and ‘Policy-induced: R&D grant (α)’ have been scaled by 1 million

Bearing the above in mind, rows two and three in Table 5 show the link between policy-induced R&D from each R&D support type, and firms' turnover, exports, and GVA. The results indicate that policy-induced R&D from R&D grants and R&D tax credits, is positively and significantly linked to higher performance outcomes of subsidiaries. These results suggest strong support for Hypothesis 3. However, we build on, and extend previous studies by focusing on the specific case of foreign-owned subsidiaries, including both main R&D support types in the same analysis, along with using a wider range of firm performance outcomes. In addition, by constructing a novel dataset which is between double and 10 times larger than those used in previous studies, we overcome many data limitations faced by previous studies which focused on foreign-owned subsidiaries.

To further test our support for Hypothesis 3, we compare the results of privately-funded R&D to those of policy-induced R&D. Focusing on the foreign-owned subsidiary sample in Table 5, as anticipated, privately-funded R&D has a positive and significant link to all firm performance measures. To interpret these results, we perform the following calculations. Firstly, the privately-funded and policy-induced R&D variables are defined in Euro-terms, while the dependent variables are given in logarithm. Therefore, we can interpret the coefficients as semi-elasticities. As such, our results for subsidiaries show that a €1,000,000 increase in private R&D relates to 0.806% higher turnover, 0.751% higher GVA, and 0.734% higher exports. These findings are in line with those reported by Kancs and Siliverstovs (2016), when reviewing the literature on the impact of private R&D on firm performance.

Secondly, we formally examine whether the two estimated coefficients for policy-induced R&D are statistically different, by performing a series of t-tests. Results show that there is no statistically significant difference at the 5% level for policy-induced and privately-funded R&D. This holds for all statistically significant coefficients of policy-induced R&D. Therefore, we find further support for Hypothesis 3, by showing that the policy-induced R&D from both R&D supports, is as effective as privately-funded R&D for subsidiaries' firm performance.

It is important to note that we observe a similar pattern of positive and statistically significant results in both the foreign-owned and domestic samples. These results suggest that foreign-owned subsidiaries do exploit the results from host country-funded R&D in the host country, at least to an extent sufficient to increase firm performance. Overall, these results reinforce our support for Hypothesis 3.

We next turn to Hypothesis 4, which states that policy-induced R&D from R&D grants has a stronger link to foreign-owned subsidiaries' performance, relative to R&D tax credits. In this regard, a further series of t-tests reveal no statistically significant difference between the coefficients of each R&D support type at the 5% level. Therefore, although the R&D induced by grants is significantly linked to subsidiary turnover, exporting, and GVA, we find no support for Hypothesis 4. In this regard, our results are similar to Nilsen et al. (2020), who find that R&D grants appear to be somewhat less effective than R&D tax credits at driving firm performance. It is important to highlight that Nilsen et al. (2020) focus on the *direct effects* of R&D support on firm performance in a general sample of firms. In contrast, our analysis focuses on the role of *policy-induced R&D* from each R&D support type for firm performance, in the specific case of foreign-owned subsidiaries. Our results suggest that the *type of R&D* stimulated by R&D grants in subsidiaries does not significantly differ from that induced by R&D tax credits. Therefore, our results build on those presented by Nilsen et al. (2020), by examining the specific case of foreign-owned subsidiaries operating in a host country.

### Robustness tests

As detailed in Sect. 3.2, we perform a series of robustness tests to examine the sensitivity of our stage two results to changes in stage one PSM model specifications, and potential policy instrument mix effects. Appendix Tables G2 and G3 in the Supplementary material accompanying this paper, present estimates obtained from tests of the robustness of our stage two model, to changes in the PSM model used in stage one. Results from these robustness tests confirm those presented in Table 5. This suggests that our stage two results are not dependent on the key policy-induced R&D variables being generated by one specific method. Table G4 in the Supplementary material accompanying this paper presents the results of our policy instrument mix robustness tests. Here, we split the policy-induced R&D variables into four categories. These capture R&D induced in situations where firms receive one policy instrument only, or a combination of both policy instruments. In almost all instances, this robustness test demonstrates strong support for our main results.

Overall, results from Table G4 in the Supplementary material accompanying this paper suggest that policy instrument mix is not the main driver of the link between R&D grants, R&D tax credits, and firm performance improvements. Policy-induced R&D in situations where firms receive R&D tax credits only, and a combination of R&D tax credits and R&D grants, have a positive and significant association with almost all firm performance measures. The main exception to this rule comes from policy-induced R&D, where firms receive an R&D grant only. In this instance, only a small number of firm performance measures are associated with grant-induced R&D. This result may be due to purely empirical factors. The sample size reduces significantly when we split the policy-induced R&D variables into four categories. This is particularly acute in the case of foreign-owned firms receiving R&D grants only, as this is our smallest sample to begin with.

An alternative explanation for the results in Table G4 in the Supplementary material accompanying this paper, as discussed in a major report by the OECD (2020), is that they could be driven by the fact that R&D grants are usually associated with more long-term and radical forms of innovation. Such potentially far-from-market R&D can take more time to drive firm performance (Smith et al., 2018). In addition, it is more likely to produce economic returns outside of the host country, in cases where the more radical innovations are commercialised by other subsidiaries of the parent firm (European Commission, 2017a). R&D tax credits, in contrast, are used more in the near-to-market and development stages of an innovation project, producing more immediate performance effects (European Commission, 2017b). Moreover, if/when R&D grants do have firm performance effects, it may be natural to expect they will be received in combination with R&D tax credits in many cases (Neicu et al., 2016). Although beyond the scope of this paper, it would be a useful avenue for future research to perform a specific analysis of policy instrument mix effects, and delve further into the mechanisms underpinning these results. Appendix Tables G5 and G6 in the Supplementary material accompanying this paper, show that our robustness tests for policy instrument mix effects presented in Table G4 in the Supplementary material accompanying this paper, are themselves robust to changes in the PSM model used in stage one.

Our final robustness test, as detailed in Sect. 3.2, is to estimate Eq. (4) utilising the Lewbel (2012) method, as an alternative approach to controlling for endogeneity. The results of the Lewbel (2012) estimation of Eq. (4) are presented in Appendix Table G7 in the Supplementary material accompanying this paper. We observe that the results confirm the robustness of the findings presented in the main paper. All policy-induced R&D expenditure variables are consistent with our main results presented in Table 5, in all models across all outcome variables (i.e. policy-induced R&D from R&D grants and R&D tax credits, in foreign-owned and domestic firms, across turnover, exports, and GVA). The sole exception to this is for R&D grant-induced R&D for foreign-owned subsidiaries, in the case of firm-level GVA, which becomes non-significant in the Lewbel model. As such, this robustness test strongly suggests that our main results in Table 5 are not biased due to endogeneity.

## Discussion and conclusions

Policymakers in many countries allocate vast sums of scarce public money to fund the R&D activities of foreign-owned subsidiaries based in their host economies (European Commission, 2017a). This is a particularly important issue in small, open economies (Appelt et al., 2016), which often pursue a strategy of attracting and embedding FDI as a means of leveraging R&D-driven firm performance improvements (OECD, 2015; Rodríguez-Pose & Wilkie, 2016). In this study, we provide, to our knowledge, the first comprehensive evaluation of the link of public R&D funding to firm performance in foreign-owned subsidiaries, considering both grant-based and tax-based R&D funding schemes. All previous analyses have been hindered by lack of data availability. We overcome this issue by constructing a unique panel dataset for Ireland, with 24,404 observations over 10 years. Moreover, based on a comprehensive review of the literature on public R&D support, we identify three issues which have not been fully addressed in previous research: (1) Disentangling the impact of publicly-funded R&D, from that of purely privately-funded R&D, on firm performance; (2) The differential impacts of R&D grants and R&D tax credits; and, (3) Examining R&D and firm performance impacts in the same analysis.

By addressing each of these points in the same analysis, we build on and advance previous studies, thus contributing to the literature on public support for firm-level R&D. Our main finding is that public funding for R&D has a strong link with foreign-owned subsidiary performance in the host country. The result holds for both R&D grants and R&D tax credits. This policy-induced improvement in foreign-owned subsidiary performance also generates economic gains for the host country that are well-aligned with the FDI policy objectives of many countries. In the Irish context (as detailed in Sect. 3.2), stimulating higher levels of R&D investments, and in situ commercialisation of publicly-supported R&D results by foreign-owned subsidiaries based in Ireland, are considered desirable policy outcomes. Thus, our findings suggest that an industrial strategy of attracting FDI to achieve R&D-linked firm performance improvements, may be worth exploring as a viable strategy for economies beyond the Irish context. The choice of policy instrument does not seem to have an important role to play in this respect. As detailed in Sect. 2.1, in situ commercialisation of foreign-owned subsidiaries’ R&D, represents an important dimension for how host countries realise payoffs from supporting foreign-owned subsidiary R&D.

By investigating these key issues, our study makes two important contributions to the literature on public funding for R&D. Our first contribution is that we examine the unique risk that foreign-owned firms may conduct R&D in a host country with the support of public funding, but not exploit the R&D results in their host country (European Commission, 2017b). Our results show that, at least to a sufficient extent, this key risk does not materialise. Both R&D grants and R&D tax credits are effective at driving subsidiaries’ R&D. Moreover, the additional R&D induced by these policy supports, translates, on average into higher performance of the subsidiaries. Our rich database also enabled our analysis to examine Antonelli’s (2020) call for a strong additionality requirement, when allocating public R&D support to private firms. Our results demonstrated that, on average, €1 of R&D tax credit support leads to less than €1 of additional R&D spending in foreign-owned subsidiaries, while R&D grant support results in more than €1 additional R&D spending in foreign-owned subsidiaries. These results suggest that, on average, R&D grant support is more effective at driving firm-level R&D in foreign-owned subsidiaries. In terms of firm performance outcomes, R&D induced both by grants and tax credits tends to have similar impacts. Moreover, the effectiveness of policy-induced R&D is statistically equal to that of purely privately-financed R&D. This suggests that public R&D support plays an equally important role in driving firm performance in foreign-owned subsidiaries, as private R&D does.

Our second contribution focuses on evaluating the relative impacts of R&D grants and R&D tax credits. In most countries, these policy instruments account for the vast majority of all public R&D funding directed at private firms (Cunningham & Link, 2021). Despite this, the relative effectiveness of these instruments at driving R&D and firm performance improvements in foreign-owned subsidiaries is completely unaddressed in previous analyses (e.g. Acheson & Malone, 2020; Giroud et al., 2012; Liu et al., 2016). In this regard, our study fills an important gap in the literature. Our results show that both R&D grants and R&D tax credits, produce a similar impact on foreign-owned subsidiaries’ R&D, which is significantly linked to firm performance improvements.

From a policy perspective, our study contributes to the debate on R&D FDI policy, and the R&D grants *versus* R&D tax credits debate more generally. We find positive input additionality for both R&D support types. We find higher additionality for grants (as compared to tax credits) in the case of foreign-owned subsidiaries, and higher additionality for tax credits (as compared to grants) in the case of domestic firms. However, in our sample, it is noteworthy that R&D tax credits account for approximately twice the monetary amount of public R&D funding *vis-à-vis* R&D grants. This situation is mirrored in several countries (Cunningham & Link, 2021; Lenihan et al., 2020). Given that each R&D support type functions through different mechanisms, the European Commission (2017b) has suggested that a lack of balance in funding amounts such as this, is potentially important from a policy perspective. R&D tax credits can help firms to engage in R&D spending which may not otherwise have been possible, but this spending is completely at the firm’s discretion (Petrin & Radicic, 2023). In contrast, R&D grants enable governments to direct firms towards specific R&D projects, which are deemed strategically important, but lack sufficient market incentives (Hud & Hussinger, 2015). As well as providing key insights for other small open economies pursuing an FDI focused development strategy (Cunningham et al., 2020), our findings may be relevant for countries beyond the Irish case. Such countries include the UK, France, and Belgium, where the level of R&D tax credit support has also grown to almost double that of R&D grants since circa 2012 (Lenihan et al., 2020). In addition, countries such as Germany, where an R&D tax credit programme has recently been launched (in 2020), could also learn from the Irish experience. Our results suggest that policymakers might usefully consider a greater balance between the use of R&D grants and R&D tax credits, as a means to achieve R&D policy goals.

It is interesting to place our results in the context of the OECD’s (2018) country report for Ireland. This report recommends that Ireland shift away from R&D tax credits, suggesting they favour foreign-owned subsidiaries, at the expense of domestic firms. Instead, the OECD suggests that Ireland should focus on direct funding for R&D in domestic firms, to build their technological capabilities. The IMF (2016) echo a similar recommendation for countries beyond the Irish case. Specifically, they highlight the benefits of targeted R&D grants for small resource constrained firms, and moreover, the challenges in designing R&D tax credit programmes which not only benefit large firms (with high levels of pre-existing R&D intensity). However, the European Commission (2017a, 2017b) and Rodríguez-Pose and Wilkie (2016) suggest the opposite—R&D tax credits can play a key role in attracting foreign R&D investment, which can have important economic impacts for host countries. Should the Irish government decide to reduce its reliance on R&D tax credits, our results suggest that a greater shift towards direct grant funding would benefit foreign-owned subsidiaries’ R&D activities. This is because grants show a much higher input additionality, compared to tax credits. However, for domestic firms, the opposite applies. While our results shed some light on these issues, it is important to note that, due to our data and model set-up, we cannot deduce that it is better to spend public money on foreign subsidiaries rather than on domestic firms (or vice versa). This is chiefly because, in our analysis, we are unable to compare foreign-owned subsidiaries to comparable domestic firms (i.e. to domestic business groups).

As Guimón et al. (2018: 167) note, “besides attracting new flows of R&D-related FDI, policy interventions should also aim at *embedding* the existing R&D activity of MNE subsidiaries in the national innovation system”. Although beyond the scope of our paper, the impact of public support for R&D on embeddedness, identified as a key dimension of potential payoffs to a host country, merits further investigation in future research. Moreover, our call for further research in this vein is supported by DJEI’s (2014a: 4) *Policy Statement on Foreign Direct Investment in Ireland*, which noted that, “[o]ther questions arise as to what policy approaches are needed to further develop inter‐firm linkages with Irish owned entities, optimise the potential arising from different modes and forms of FDI, and FDI’s contribution to job creation and regional development”. It is important to note that this statement was considered significant enough, from the perspective of a country with an intensive policy focus on foreign-owned subsidiaries’ R&D (i.e. Ireland; see Cunningham et al., 2020) to place in a major policy document. However, to the best of our knowledge, no previous research has examined the impact of public R&D support on foreign-owned subsidiaries’ embeddedness, thus suggesting an area for future investigation.

Although our study makes significant contributions to the literature on public funding for R&D, it is not without limitations. Beyond the data limitations discussed in Sect. 3.3, we note further limitations that could be fruitfully addressed in future research. Firstly, we focus only on direct firm-level impacts. Engel et al. (2019) highlight that indirect effects such as spillovers, may influence direct impacts. Whilst beyond the scope of the current study, future research is necessary to examine such issues. Secondly, our study is not in a position to comment on the marginal returns to R&D. This will depend to a large extent on the type of R&D firms conduct (e.g. basic research, applied research, and development; developing new technologies *versus* improving existing technologies), as opposed to how much firms spend on R&D (Mulligan et al., 2022; Neicu et al., 2016). Thirdly, our theoretical background, model set-up choices, and results discussion have focused solely on foreign-owned subsidiaries. This is due to the key gap in the literature identified in this regard. However, our results also reveal important effects in the case of domestically-owned firms. Therefore, future research would benefit from a dedicated analysis of domestic firms (and in the context of other countries, beyond the Irish case).

As discussed in Sect. 3.1, although our dataset is large and rich, it does not contain a variable identifying whether domestic firms are part of a domestic business group, or are themselves domestic multinational firms, with subsidiaries based outside of Ireland. Future analysis of domestic firms would benefit from a detailed examination as to whether business group structure influences how public R&D support drives firm-level outcomes. In terms of data limitations, despite the unprecedented richness of our dataset, it was not possible to assess the specific effects of all the knowledge bases (e.g. patents, co-operation agreements with clients, customers and other firms) available to foreign-owned subsidiaries locally, and within the global MNE network. Notwithstanding this limitation, the use of a fixed effects modelling approach enables us to capture many unobserved effects. However, future research (and given the available data) would benefit from explicitly capturing important factors of firms’ knowledge bases, such as those outlined above. Finally, it is important to highlight that our analysis can only claim to examine the correlation between policy-induced R&D and firm performance impacts, as opposed to causal effects. Future research would benefit from the implementation of a causal effects analysis, should data availability facilitate this. As detailed by De Blasio et al. (2015) and Lanahan (2016), this type of methodological advancement requires a sophisticated type of instrumental variable analysis. Despite its vast scale, and to a large degree unprecedented richness *vis-à-vis* previous studies which focus on foreign-owned subsidiaries in a host country, this is not possible with our dataset. Notwithstanding these limitations, by focusing on the specific case of foreign-owned subsidiaries based in a host country, our study makes significant contributions, and represents an important step forward to the literature on public funding for R&D.

## Supplementary Information

Below is the link to the electronic supplementary material.

Supplementary file1 (DOCX 157 KB)

## Footnotes

1 Based on correspondence between the authors and the Department of Business, Enterprise and Innovation, we can infer that all the foreign-owned firms in our dataset are subsidiaries of multinational firms.

2 As is common in previous studies (for a discussion, see Kwon and Park, 2018), it is not possible for us to identify domestically-owned firms who have their own subsidiaries based outside of Ireland, and who potentially exploit R&D abroad. Although some such firms exist, they constitute a small fraction of the domestic sample (see: https://enterprise.gov.ie/en/Publications/ABSEI-2019.html).

3 Correspondence with the Irish Department of Enterprise, Trade and Employment (DETE), confirmed that in the ABSEI dataset, it is not possible to identify domestic firms which belong to a group in a usable way for our analysis.

4 We use the European Union recommendation 2003/361 which defines small-sized firms as firms with less than 50 employees, medium-sized firms as firms with at least 50 and fewer than 249 employees, and large firms as firms with at least 250 employees. The recommendation also classifies firms according to their turnover or balance sheet (see http://data.europa.eu/eli/reco/2003/361/oj), but the number of employees is the most used classification (Eurostat, 2019). We define micro firms as those employing less than 10 employees.

5 Some previous studies which examine the impact of policy-induced R&D/innovation on firm performance also include a one-year-lagged value of the dependent variable as a control variable in their stage two analysis (see e.g. Aerts, 2008; Freel et al., 2019). We do not include such a variable in our analysis. The rationale for this decision is detailed in Appendix B in the Supplementary material accompanying this paper.

6 While this approach is commonly adopted, Hill et al. (2021) warn that time separation may not fully solve this problem unless u has zero autocorrelation. Hill et al. (2021) note that *x* at time *t* is not caused by *u* at time *t + 1*. This is because it cannot cause something in the past. Therefore, *u* at time *t + 1* will frequently be correlated with *u* in time *t*, and, as noted previously, *x* at time *t* is correlated with *u* at time *t*.

7 For more information on the policy context of Ireland and our dataset, see Appendix A in the Supplementary material accompanying this paper.

8 Full details on the sampling strategy for ABSEI can be found in Appendix D in the Supplementary material accompanying this paper.

9 Further details on the specific R&D grant programmes included are provided in Appendix Table A1 in the Supplementary Material accompanying this paper.

10 This variable does not comprise direct R&D grant funding to firms, but rather includes supports designed to enable collaboration with the higher education system (see Appendix A in the Supplementary material accompanying this paper).

11 We note that it is not possible to control for foreign-owned subsidiaries’ country of origin in our analysis.

12 We note Cozzarin and Percival (2021) use training in per employee terms.

13 As a robustness test, we have also performed this analysis using a bivariate probit model. The results from this model mirror our logit model results, as do the follow-on stage one and stage two results based on the bivariate probit model propensity scores. These results are available from the corresponding author on request.

14 We note that if foreign-owned subsidiaries do not commercially exploit host-country-funded R&D in the host country, their parent firm may exploit it at other locations in their global network, or they may not exploit it at all. Our study has no measure for the global returns to host country-funded R&D. We can only examine whether firm performance benefits accrue in the host country.

15 For a more detailed discussion of crowding-out effects in a two-stage model, see Beck et al. (2016).
