# Supplementary material for: R&D grants and R&D tax credits to foreign-owned subsidiaries: Does supporting multinational enterprises’ R&D pay off in terms of firm performance improvements for the host economy?
Source: J Technol Transf. 2023 Feb 13;49(2):740–81. doi: 10.1007/s10961-023-09995-9 (PMC11636753; doi:10.1007/s10961-023-09995-9)
Supplement: Supplementary file 1 — Supplementary file1 (DOCX 157 KB) [file 10961_2023_9995_MOESM1_ESM.docx]

**Supplementary material**

**Appendices to the article:** **“R&D grants and R&D tax credits to foreign-owned subsidiaries: Does supporting Multinational Enterprises' R&D pay off in terms of firm performance improvements for the host economy?”**

**Appendix A: Policy context of Ireland**

Examining the link between public R&D support, and the R&D and economic performance of foreign-owned subsidiaries is particularly relevant in Ireland, given the duality (in terms of foreign-owned and domestically-owned firms) of the Irish economy (OECD, 2018; Cunningham et al., 2020). The Irish economy is characterised by a large presence of foreign-owned firms in certain key high-technology sectors such as electronics, pharmaceuticals, software, and international services^[[1]](#footnote-2)^. It is widely acknowledged that foreign-owned firms have had a transformative effect on the Irish economy (DBEI, 2014; OECD, 2018). For example, Figure A1 below shows that over the period 2007-2018, foreign-owned firms accounted for two-thirds of Ireland's total business expenditure on R&D^[[2]](#footnote-3)^. Since 1949, Ireland has had a specific funding agency responsible for attracting FDI into Ireland: Industrial Development Agency (IDA) Ireland.

Notwithstanding the above, the Irish government’s continued pursuit of an FDI-led growth model has also been questioned over several decades for leaving the economy vulnerable to external shocks, coupled with what some commentators regard as a neglect of domestic Irish firms (OECD, 2018; Bailey and Lenihan, 2015; Cunningham and Golden, 2015). Following recommendations on the development of a strong domestic industrial base, Enterprise Ireland (EI) was created in 1998 to provide tailored support for domestically-owned firms. EI provides a range of different R&D/innovation supports to domestic firms, while IDA focuses on a smaller number of large R&D supports targeted at foreign-owned firms. Over the period 2007-2016, 68 per cent of foreign-owned firms in Ireland supported by IDA Ireland originated from the USA^[[3]](#footnote-4)^. Finally, in terms of financial support for R&D, the R&D tax credit is by far the largest form of R&D support in Ireland^[[4]](#footnote-5)^, in terms of the number of firms who claim it, and the amount of public funding dedicated through it (Lenihan et al., 2020; Cunningham and Link, 2021). The Irish R&D tax credit provides a 25 per cent refund on qualifying R&D expenditures, and is available to both domestic and foreign-owned firms undertaking qualifying R&D activity in Ireland^[[5]](#footnote-6)^.

While the number of foreign-owned firms supported by IDA is significantly lower relative to the domestic firms supported by EI, the scale of funding that IDA provides is significantly higher. In our sample, (detailed in Table A1 below), IDA allocated approximately €501 million in direct funding for R&D, in contrast to €241 million for EI. This highlights that a much smaller number of foreign-owned firms are in receipt of larger R&D grants, as well as the fact EI provides a much wider range of R&D supports, meaning its funding is more dispersed. Foreign-owned firms are also by far the largest recipient of R&D tax credits (see Table A2 below). Indeed, the OECD (2018) has recently recommended that Ireland move away from the use of R&D tax credits, which it suggests mainly benefit foreign-owned firms, and focus more on R&D grants, targeted at building domestic firms' technological capabilities. However, it is crucial to determine whether supports such as the R&D tax credit are producing an economic return for the Irish economy (a key component of which culminates in firm level performance benefits) before such recommendations are implemented.

Another factor which may directly influence firms' R&D expenditure is collaboration with Higher Education Institutions (HEIs) facilitated by Irish government policy programmes. Several such programmes exist in Ireland, but *do not* involve direct R&D grant payments, or tax incentives to conduct R&D. The main schemes available to firms in Ireland are the Innovation Vouchers programme and the Innovation Partnerships programme, which are implemented by EI, but can also be availed of by IDA client firms.^[[6]](#footnote-7)^ In addition, Science Foundation Ireland (SFI), a separate national funding agency, implements a research centres programme available to both domestic and foreign-owned firms. SFI research centres do not provide R&D funding directly to firms, but rather provide access to world-leading scientific knowledge through research collaborations between research centres located at HEIs and firms.^[[7]](#footnote-8)^ Although the focus of our study is on R&D grants and R&D tax credits, we nevertheless control for the presence of these other R&D supports, as they too are designed to influence firm-level R&D. Also, by including these other supports, our study captures the full/holistic system of firm-level R&D supports in Ireland.

As discussed in Section 2.1 in the main paper, which reviewed the literature on R&D internationalisation, the concept of embeddedness is one important dimension when considering how R&D in foreign-owned subsidiaries leads to payoffs for the host economy (Belitz and Mölders, 2016; Papanastassiou et al., 2020). Our study focuses on another important dimension: The *in situ* commercialisation of publicly-supported R&D by foreign-owned subsidiaries (i.e. the commercialisation at the host country location, as opposed to commercialisation activities at other locations of the parent MNE). In the specific context of Ireland, repeated policy documents and assessments from the Irish government, European Commission, and OECD have made clear that attracting R&D activities of foreign MNEs to Ireland, and increasing the R&D expenditures of foreign-owned subsidiaries already located in Ireland, is a crucial pillar of national economic competitiveness (European Commission, 2007; DJEI, 2014a; 2014b; OECD, 2020). As such, public R&D support targeted at foreign-owned firms is a major part of Irish industrial policy (DJEI, 2014b; OECD, 2020). Moreover, the foreign-owned subsidiaries’ commercialisation of publicly-supported R&D, which takes place *in situ* in Ireland, has long been established as a key policy goal (European Commission, 2007; DJEI, 2014a; 2014b; DFHERIS, 2022). This policy goal is concisely expressed in DJEI’s (2014a: 10) *Policy Statement on Foreign Direct Investment in Ireland*, which notes the need to “[t]arget FDI that can commercialise and exploit in Ireland those areas prioritised for research investment”.

An earlier report from the European Commission (2007) preceded the Irish Government’s own policy emphasis on *in situ* commercialisation of R&D, by foreign-owned subsidiaries. This report (p. 64) highlighted two important features of R&D internationalisation on the global scale: 1) The “growing tendency towards the de-linking of the place where the commercial exploitation of the outcomes of R&D takes place with the place where the R&D is performed”; and 2) That this phenomenon will “enhance policy makers to (consider to) intervene in the process of the internationalisation of the exploitation of the knowledge results stemming from research”. These points have again been emphasised in more recent reports from the European Commission (2017a; 2017b). In the specific case of Ireland, The European Commission (2007: 67) noted that “[i]n terms of the exploitation and commercialisation of research performed in Ireland, there is a strong emphasis on capturing the economic benefits of (publicly-funded) research within the island”. On this point, the OECD’s 2020 *FDI Qualities Assessment of Ireland* report concludes that “[c]ontinuing to seek new investments in technology-intensive activities, and enhancing R&D activities in Ireland, is likely to be successful in the future and to contribute to economic and productivity growth in Ireland” (p. 10).

In a major assessment of public R&D support available to foreign-owned subsidiaries based in Ireland, DJEI (2014b: 289) specifically sought to gauge “how delivering the R&D project has supported the performance of the company in Ireland”. The results of this assessment are striking: 88% of foreign-owned subsidiaries agreed that the “company is now more embedded in Ireland as a result of the R&D project supported by the IDA” (DJEI, 2014b: 292). In addition, 60% reported turnover would be lower if the project had not gone ahead, and over a third of respondents indicated they may not still be located in Ireland without the supported R&D project. Indeed, the IDA’s own R&D funding guidelines highlight *in situ* commercialisation of publicly-supported R&D as a key funding objective, stating the following: “Specifically IDA Ireland envisages that companies utilising this RD&I grant aid will be aiming towards achieving … demonstrable connection between R&D activity and commercial outputs”.^[[8]](#footnote-9)^ Moreover, the recent (Department of Further and Higher Education, Research, Innovation and Science (DFHERIS; 2022: 7) strategy document, *Impact 2030: Ireland’s Research and Innovation Strategy*, makes clear that “an acceleration of research commercialisation” remains a key policy goal, for both domestic firms and foreign-owned subsidiaries based in Ireland.

**Figure A1:** Firm-level expenditure on R&D in Ireland (2007-2018), by firm ownership


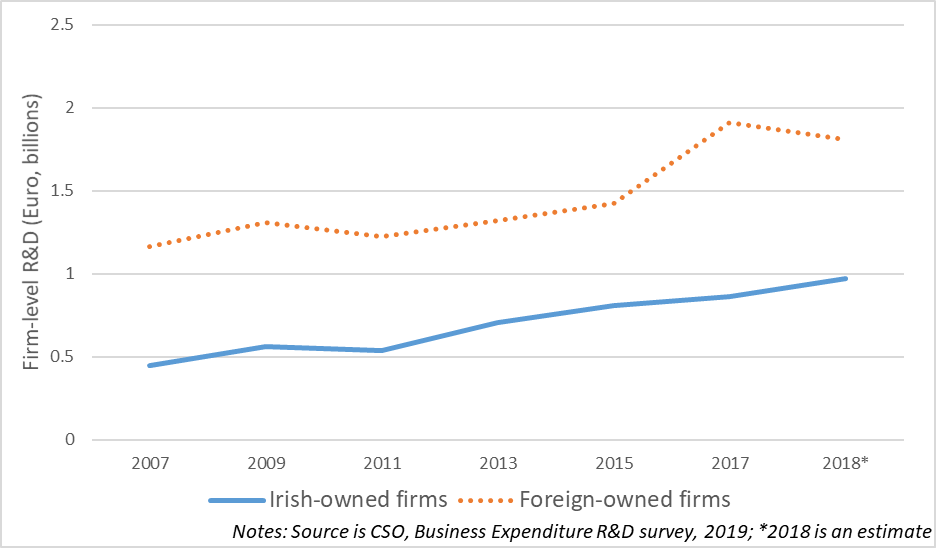


| **Table A1:** Direct funding programmes for R&D allocated to foreign-owned and domestic firms based in Ireland (2007-2016) | | |
| --- | --- | --- |
|  | *Foreign-owned firms* | |
| IDA Ireland funding programme name | Number of grants allocated | Euro value of funding (millions) |
| R&D Innovation | 301 | € 483.41 |
| RD&I Feasibility | 55 | € 7.64 |
| R&D Capability | 7 | € 10.47 |
| Total | 363 | € 501.51 |
|  | *Domestically-owned firms* | |
| Enterprise Ireland funding programme name | Number of grants allocated | Euro value of funding (millions) |
| R&D fund | 1,099 | € 156.59 |
| Innovative High Potential Start-Up (HPSU) fund | 216 | € 52.23 |
| Company Expansions including R&D | 97 | € 23.60 |
| Intellectual Property Assistance Scheme (IPAS) | 7 | € 0.10 |
| Technical Feasibility grant | 418 | € 9.33 |
| Total | 1,837 | € 241.87 |
| **Notes:** Direct funding programmes for foreign-owned firms are implemented by IDA Ireland, while direct funding programmes for domestically-owned firms are implemented by Enterprise Ireland. This table is based on the merged IDA Ireland and Enterprise Ireland administrative data and the Annual Business Survey of Economic Impact (ABSEI) used in our analysis, not the full population of R&D grant recipients. See Appendix E for descriptive statistics on the full IDA Ireland and Enterprise Ireland data, and representativeness tests against our merged sub-sample. | | |

| **Table A2:** R&D tax credit claims by foreign-owned and domestic firms based in Ireland (2007-2016) | | | |
| --- | --- | --- | --- |
|  | Claims by foreign-owned firms |  | Claims by domestically-owned firms |
| Total amount (approximate) | €1,004,607,500 |  | €473,847,500 |
| Total claims | 989 |  | 3,710 |
| **Notes:** This table is based on the merged Irish Revenue Commissioners R&D tax credit claims data and the Annual Business Survey of Economic Impact (ABSEI), not the full population of R&D tax credit claimants. The full population data does not contain an indicator of whether firms are foreign-owned, and therefore cannot be used to split the sample based on ownership type. See Appendix Tables E3 for representativeness tests of our merged sub-sample against the full sample. The figure for total amount is an approximation. This is because the Irish Revenue Commissioners and Central Statistics Office (CSO) provide the R&D tax credit claim data in Euro amount bands (16 categories, ranging from €1 to €25,000, to Greater than €10 million), rather than actual Euro amounts. The Irish Revenue Commissioners and CSO provide the data in this way to anonymise individual claims. However, the individual claim bands are deemed to be disclosive under the CSO's policy on Statistical Disclosure Control. Therefore, we present the approximate total claims for foreign-owned firms and domestic firms, rather than claims in each band. The approximate total is calculated based on the mid-point of each claim band (the value of €15 million is used for the top claim band, as it is open ended). | | | |

**Appendix B: Lagged dependent variables**

Some previous studies which examine the impact of policy-induced R&D/innovation on firm performance also include a one-year-lagged value of the dependent variable as a control variable in their stage two analysis (see e.g. Aerts, 2008; Cin et al., 2017; Freel et al., 2019). We do not include such a variable in our analysis for two reasons. Firstly, Allison et al. (2017) argue that including lagged dependent variables in dynamic panel models (using fixed or random effects) is a major source of estimation bias. In this type of model, the constant term represents the combined effect of all time-invariant unobserved variables on the dependent variable. A key assumption underpinning this process is that it is not influenced by the independent variables included in the model. However, Allison et al. (2017) detail that lagged dependent variables (i.e. in time t-1) interact with all unobserved time-invariant factors that impact the dependent variable in time t. Including such a variable therefore, violates a key underpinning assumption necessary for reliable estimation. Secondly, as discussed by Cin et al. (2017), correcting for the bias introduced by including a lagged dependent variable in our type of empirical set-up would require the implementation of a Generalised Method of Moments (GMM) model. Implementing a GMM model requires a minimum of three consecutive observations on each firm in the dataset to generate the instrumental variables necessary for accurate estimation. Although our sample size is large, we are using an unbalanced panel dataset. Therefore, the requirements of the GMM model would reduce our sample size by 37 percent, severely impacting the predictive power and representativeness of our analysis. For these reasons, we chose not to include lagged dependent variables in our stage two estimations. Instead, we include firm and year fixed effects, and a series of time varying control variables which previous studies (e.g. Freel et al., 2019) indicate are associated with firm performance.

**Appendix C: Variable definitions and descriptive statistics**

In addition to defining and providing descriptive statistics for our key variables (in Tables C1 and C2 below respectively), here we also provide additional details on how our treatment variables (R&D tax credits and R&D grants) are measured in the analysis. For the R&D tax credit, we define a firm as treated in the year before it files an official R&D tax credit claim. The rationale for measuring the R&D tax credit in this way is that the actual R&D spending which necessitates the claim takes place in the year before the claim is filed. The timing here is important because any policy-induced R&D will pre-date the actual claim by one year. For the R&D grant, we measure the treatment variable as any year firms received a direct financial payment from Enterprise Ireland or IDA Ireland. In many countries, firms are often approved for R&D grants in a specific year, and they draw down on this grant funding over the following years. Therefore, one grant can be associated with several individual financial payments to the firm over many years. As a result, our definition of the R&D grant variable helps to ensure that we capture the link between policy-induced R&D in each year that firms receive direct R&D funding. Defining the R&D grant variable in this way is only possible because we have detailed administrative data on R&D grant allocations.

| **Table C1:** NACE codes and description of NACE sectors included in the analysis | |
| --- | --- |
| NACE codes | Description of sector |
| 1-12 | Food, Drink & Primary Production |
| 13-18, 22-25, 27-33 | Traditional Manufacturing |
| 19-21 | Chemicals |
| 26 | Computer, electronic & optical products |
| 34 | Medical devices |
| 35-43 | Energy, water, waste & construction |
| 45-56, 68-96 | Business, education & other services |
| 58-61 | Publishing, broadcasting & telecommunications |
| 62-62.01 | Computer programming |
| 62.02 | Consultancy & related facilities |
| 62.03-63 | Other Information Technology and computer services |
| 64-66 | Financial services |
| **Notes:** NACE is the acronym for 'nomenclature statistique des activités économiques dans la Communauté européenne', and is the statistical classification of economic activities used by Eurostat. Sector descriptions are those specified in DETE's (2021) report on the ABSEI survey, which uses NACE Rev. 2 classifications (see: <https://www.cso.ie/en/qnhs/qnhsmethodology/naceclassificationslist/>). | |

| **Table C2:** Descriptive statistics in full sample and treatment sub-samples | | | | | | | |
| --- | --- | --- | --- | --- | --- | --- | --- |
|  | Full sample | Foreign-owned firms | Domestic firms | R&D grant recipients,  foreign | R&D grant recipients, domestic | R&D tax credit recipients,  foreign | R&D tax credit recipients, domestic |
| Variables | Mean (SD) | Mean (SD) | Mean (SD) | Mean (SD) | Mean (SD) | Mean (SD) | Mean (SD) |
| R&D tax credit (1/0) | 0.192 (0.394) | 0.184 (0.387) | 0.196 (0.397) | 0.432 (0.495) | 0.443 (0.496) | \ | \ |
| R&D grant (1/0) | 0.110 (0.313) | 0.081 (0.273) | 0.105 (0.307) | \ | \ | 0.190 (0.392) | 0.239 (0.427) |
| R&D (log) | 7.746 (6.065) | 7.682 (7.046) | 7.759 (5.760) | 14.34 (3.720) | 11.22 (3.800) | 13.60 (3.455) | 12.23 (2.645) |
| R&D above median (1/0) | 0.675 (0.468) | 0.729 (0.444) | 0.659 (0.473) | 0.707 (0.455) | 0.589 (0.492) | 0.471 (0.499) | 0.266 (0.442) |
| Turnover quartile one (1/0) | 0.230 (0.421) | 0.226 (0.418) | 0.231 (0.421) | 0.141 (0.349) | 0.282 (0.450) | 0.133 (0.339) | 0.241 (0.428) |
| Turnover quartile two (1/0) | 0.251 (0.433) | 0.251 (0.434) | 0.251 (0.433) | 0.157 (0.365) | 0.220 (0.414) | 0.253 (0.434) | 0.235 (0.424) |
| Turnover quartile three (1/0) | 0.257 (0.437) | 0.261 (0.439) | 0.256 (0.436) | 0.194 (0.396) | 0.251 (0.433) | 0.244 (0.430) | 0.250 (0.433) |
| Turnover quartile four (1/0) | 0.260 (0.439) | 0.260 (0.438) | 0.260 (0.439) | 0.505 (0.500) | 0.245 (0.430) | 0.368 (0.482) | 0.271 (0.444) |
| Turnover (log) | 8.482 (1.978) | 10.20 (1.962) | 7.987 (1.687) | 11.32 (2.201) | 7.778 (1.837) | 10.77 (1.924) | 8.023 (1.728) |
| Exports (log) | 7.566 (2.540) | 9.975 (2.174) | 6.800 (2.140) | 11.25 (2.274) | 6.889 (2.146) | 10.62 (2.125) | 7.259 (1.999) |
| Gross Value Added (log) | 7.769 (1.831) | 9.427 (1.839) | 7.290 (1.527) | 10.59 (2.172) | 7.207 (1.592) | 9.945 (1.835) | 7.413 (1.521) |
| Past public R&D funding (1/0) | 0.355 (0.478) | 0.336 (0.472) | 0.362 (0.480) | 0.958 (0.198) | 0.867 (0.339) | 0.872 (0.333) | 0.873 (0.332) |
| Other R&D support (1/0) | 0.093 (0.291) | 0.079 (0.270) | 0.097 (0.297) | 0.247 (0.431) | 0.291 (0.454) | 0.163 (0.369) | 0.259 (0.438) |
| Micro firm size (1/0) | 0.174 (0.379) | 0.038 (0.175) | 0.215 (0.411) | 0.004 (0.067) | 0.233 (0.423) | 0.008 (0.089) | 0.198 (0.398) |
| Small firm size (1/0) | 0.479 (0.499) | 0.287 (0.452) | 0.534 (0.498) | 0.121 (0.326) | 0.531 (0.499) | 0.176 (0.381) | 0.533 (0.498) |
| Medium firm size (1/0) | 0.256 (0.436) | 0.424 (0.494) | 0.206 (0.405) | 0.343 (0.475) | 0.197 (0.398) | 0.456 (0.498) | 0.226 (0.418) |
| Large firm size (1/0) | 0.090 (0.286) | 0.256 (0.436) | 0.042 (0.202) | 0.530 (0.499) | 0.037 (0.191) | 0.358 (0.479) | 0.042 (0.200) |
| Material costs (log) | 0.956 (35.65) | 0.655 (5.928) | 1.043 (40.37) | 0.747 (2.620) | 1.290 (16.08) | 0.622 (1.759) | 1.173 (13.54) |
| Unit labour costs (log)  Training (log)  Regional R&D (log) | 1.173 (8.710)  2.381 (1.715)  18.79 (1.795) | 0.905 (3.734)  3.327 (1.870)  18.90 (1.647) | 1.249 (9.698)  2.113 (1.568)  18.77 (1.830) | 1.058 (4.377)  4.770 (1.813)  19.31 (1.334) | 1.822 (15.37)  2.349 (1.568)  19.00 (1.791) | 0.907 (3.447)  4.019 (1.714)  18.92 (1.580) | 1.600 (11.64)  2.415 (1.552)  19.09 (1.751) |
| Observations | 24,404 | 5,388 | 18,920 | 437 | 2,005 | 992 | 3,710 |
| **Notes:** NACE sectors included but not displayed; for a description see Appendix B1. The acronym SD stands for 'Standard Deviation'. | | | | | | | |

**Appendix D: Sample frame for Annual Business Survey of Economic Impact (ABSEI)**

The *ABSEI Survey Information Booklet* (DBEI, 2018) details that the sampling base covers the client base of all the Irish enterprise development agencies, and the population comprises all manufacturing and internationally traded services firms in Ireland. The population covered by the survey is compiled annually from the client base of the development agencies. In terms of sampling strategy, each of the enterprise agencies provides the Department of Enterprise, Trade and Employment (DETE) with a list of clients, who are then included in the survey (DBEI, 2018). To ensure that ABSEI is based on an up-to-date population of firms, DETE's Annual Employment Survey (AES), which provides a complete listing of establishments within the remit of each agency, is used as a sample base (DBEI, 2018). To ensure that the responding firms are representative of all firms in the AES population, weighting strata are applied, based on ownership, sector, size, and region. This design feature of ABSEI leaves it ideally placed to investigate the impact of R&D support on foreign-owned subsidiaries based in Ireland (DBEI, 2018).

**Appendix E: Sample representativeness**

| **Table E1**: Sample representativeness based on firm characteristics in the IDA Ireland data | | | |
| --- | --- | --- | --- |
|  | (a) Mean full population | (b) Mean matched sub-sample | Column (a) minus (b) |
| Funding amount (log) | 13.2 | 13.17 | 0.03 |
| Number of grants received | 1.423 | 1.501 | -0.078 |
| Owner location: USA | 0.678 | 0.706 | -0.028 |
| Owner location: Other | 0.322 | 0.294 | 0.028 |
| Region: Dublin | 0.375 | 0.281 | 0.094*** |
| Region: Mid-east | 0.056 | 0.061 | -0.005 |
| Region: Mid-west | 0.1 | 0.107 | -0.007 |
| Region: Midlands | 0.023 | 0.023 | 0 |
| Region: North-east | 0.015 | 0.017 | -0.002 |
| Region: North-west | 0.03 | 0.029 | 0.001 |
| Region: South-east | 0.065 | 0.081 | -0.016 |
| Region: South-west | 0.199 | 0.235 | -0.036 |
| Region: West | 0.133 | 0.162 | -0.029 |
| Sector: High-tech manufacturing | 0.253 | 0.305 | -0.052* |
| Sector: Medium high-tech manufacturing | 0.199 | 0.247 | -0.048* |
| Sector: Medium low-tech manufacturing | 0.048 | 0.061 | -0.013 |
| Sector: Low-tech manufacturing | 0.034 | 0.04 | -0.006 |
| Sector: Knowledge intensive services | 0.433 | 0.325 | 0.108*** |
| Sector: Less knowledge intensive services | 0.03 | 0.02 | 0.01 |
| Observations (2007-2016) | 756 | 376 | 49.74% |
| **Notes:** * p < 0.1; ** p < 0.05; *** p < 0.01. The variable 'Owner location: Other' captures all foreign-owned firms where the parent firm was not located in the USA. This aggregation is necessary because the majority of foreign-owned firms' home country is the USA. For the representativeness tests, we classify the IDA Ireland sector variable using Eurostat's six manufacturing and services industry sector aggregations (see: [https://ec.europa.eu/eurostat /cache/metadata/Annexes/htec_esms_an3.pdf](https://ec.europa.eu/eurostat/cache/metadata/Annexes/htec_esms_an3.pdf)). Our rationale for classifying the sector variable in this way is that IDA does not use a standard sectoral classification of their client firms (e.g. NACE). The large group of sectors, and low observation numbers in each sector, means that aggregating the sectors is necessary. Because IDA does not use standard sector classifications, we must aggregate the IDA sectors based on how similar the name of each IDA sector is to those which compose the EU aggregations. Therefore, we cannot assume the companies within each IDA sector fall into each EU sector aggregation as we propose, and we must treat the IDA sector representativeness tests as an approximate estimation. However, this method provides a good estimate of which IDA sectors fit into each EU aggregation, because the majority of the IDA sector names are clearly similar to those used in the EU aggregations. Importantly, this method of aggregation is the only available avenue to this study of estimating whether our sample is (approximately) representative of the full population of IDA client companies on the basis of sector. | | | |

| **Table E2**: Sample representativeness based on firm characteristics in the Enterprise Ireland data | | | |
| --- | --- | --- | --- |
| Variable | (a) Mean full population | (b) Mean matched sub-sample | Column (a) minus (b) |
| Funding amount (log) | 11.81 | 11.77 | 0.04 |
| Number of grants received | 1.47 | 1.542 | -0.072** |
| Region: Border | 0.084 | 0.103 | -0.019* |
| Region: Midlands | 0.036 | 0.037 | -0.001 |
| Region: West | 0.081 | 0.073 | 0.008 |
| Region: Dublin | 0.449 | 0.406 | 0.043** |
| Region: Mid-east | 0.076 | 0.087 | -0.011 |
| Region: Mid-West | 0.095 | 0.105 | -0.01 |
| Region: South East | 0.065 | 0.063 | 0.002 |
| Region: South West | 0.116 | 0.126 | -0.01 |
| Sector: High-tech manufacturing | 0.335 | 0.288 | 0.047*** |
| Sector: Medium high-tech manufacturing | 0.157 | 0.182 | -0.025* |
| Sector: Medium low-tech manufacturing | 0.055 | 0.065 | -0.01 |
| Sector: Low-tech manufacturing | 0.08 | 0.077 | 0.003 |
| Sector: Knowledge intensive services | 0.352 | 0.37 | -0.018 |
| Sector: Less knowledge intensive services | 0.021 | 0.018 | 0.003 |
| Observations (2007-2016) | 2670 | 1202 | 45.02% |
| **Notes:** * p < 0.1; ** p < 0.05; *** p < 0.01. For the representativeness tests, we classify the Enterprise Ireland (EI) sector variable using Eurostat's six manufacturing and services industry sector aggregations (see: [https://ec.europa.eu/eurostat/cache/metadata/Annexes/htec_esms _an3.pdf](https://ec.europa.eu/eurostat/cache/metadata/Annexes/htec_esms_an3.pdf)). Our rationale for classifying the sector variable in this way is that EI does not use a standard sectoral classification of their client firms (e.g. NACE). Over the period of this analysis (2007-2016), there are 95 individual EI sectors, 32 of which have less than 10 observations. The large group of sectors, and low observation numbers in each sector, means that aggregating the sectors is necessary. Because EI does not use standard sector classifications, we must aggregate the EI sectors based on how similar the name of each EI sector is to those which comprise the EU aggregations. Therefore, we cannot assume the firms within each EI sector fall into each EU sector aggregation as we propose, and we must treat the EI sector representativeness tests as an approximate estimation. However, this method provides a good estimate of which EI sectors fit into each EU aggregation, because the majority of the EI sector names are clearly similar to those used in the EU aggregations. Importantly, this method of aggregation is the only available avenue to this study of estimating whether our sample is (approximately) representative of the full population of EI client companies on the basis of sector. | | | |

| **Table E3**: Sample representativeness based on firm characteristics in the Revenue Commissioners R&D tax credit data | | | |
| --- | --- | --- | --- |
|  | (a) Mean full population | (b) Mean matched sub-sample | Column (a) minus (b) |
| Region: Border | 0.071 | 0.064 | 0.007 |
| Region: West | 0.101 | 0.104 | -0.003 |
| Region: Mid-west | 0.08 | 0.082 | -0.002 |
| Region: South-east | 0.058 | 0.057 | 0.001 |
| Region: South-west | 0.158 | 0.155 | 0.003 |
| Region: Dublin | 0.377 | 0.379 | -0.002 |
| Region: Mid-east | 0.104 | 0.106 | -0.002 |
| Region: Midlands | 0.046 | 0.05 | -0.004 |
| Sector: High-tech manufacturing | 0.067 | 0.082 | -0.015*** |
| Sector: Medium high-tech manufacturing | 0.101 | 0.115 | -0.014** |
| Sector: Medium low-tech manufacturing | 0.054 | 0.059 | -0.005 |
| Sector: Low-tech manufacturing | 0.199 | 0.208 | -0.009 |
| Sector: Knowledge intensive services | 0.498 | 0.466 | 0.032*** |
| Sector: Less knowledge intensive services | 0.078 | 0.066 | 0.012** |
| Firm size: Micro | 0.272 | 0.187 | 0.085*** |
| Firm size: Small | 0.386 | 0.417 | -0.031*** |
| Firm size: Medium | 0.261 | 0.311 | 0.05*** |
| Firm size: Large | 0.082 | 0.085 | -0.003 |
| Age | 18.39 | 20.007 | -1.617*** |
| Observations (2007-2016) | 8,048 | 4,858 | 60.36% |
| **Notes:** * p < 0.1; ** p < 0.05; *** p < 0.01. This table is based on the Irish Revenue Commissioners R&D tax credit data and the Central Statistics Office's Business Demography Database (BDD). All R&D tax credit claim variables come from the Irish Revenue Commissioners. All other variables (i.e. region, sector, size, age) come from the BDD. It is possible to merge the Revenue Commissioners data and the BDD based on a unique firm identifier number present in both datasets (this variable is not present in the IDA Ireland or Enterprise Ireland data, presented in Tables E1 and E2). For the representativeness tests, we classify the sector variable using Eurostat's six manufacturing and services industry sector aggregations (see: [https://ec.europa.eu/eurostat /cache/metadata/Annexes/htec_esms_an3.pdf](https://ec.europa.eu/eurostat/cache/metadata/Annexes/htec_esms_an3.pdf)), based on NACE Rev. 2 sector classifications obtained from the CSO's BDD. It is not possible to use age as a control variable in our main analysis, because this information is only present in the BDD, not the ABSEI survey. We also test for representativeness based on firms’ R&D tax credit claim amounts, using 16 claim bands provided by the CSO, which range from €1-€25,000 to greater than €10 million. These tests reveal that our sample is representative across 14 of the 16 claim bands. The only exceptions are that our sample is slightly under-represented in two claim categories: €1 million-€5 million, and greater than €10 million. We do not report the results for these tests here, because they are deemed to be disclosive under the CSO’s policy on statistical disclosure control. | | | |

**Appendix F: Propensity score matching analysis**

Table F1 below presents the results from our logit models, estimating firms’ propensity to receive a treatment. For ease of interpretation, Table 2 in the main paper presents the marginal effects from the coefficients in Table F1. In line with previous studies (e.g. Czarnitzki and Lopes-Bento, 2013; Hud and Hussinger, 2015; González and Pazó, 2008), our results demonstrate that higher levels of pre-treatment R&D, and previous public R&D funding have a major influence on whether firms receive R&D tax credits or R&D grants. While these results are common for both types of R&D support and both firm ownership types, Table F1 also reveals several notable differences. Focusing first on foreign-owned firms, it is clear that past turnover plays a key role in determining whether subsidiaries claim R&D tax credits, but has little impact on the likelihood of receiving R&D grants. In contrast, when examining domestic firms, past turnover has no significant impact on the likelihood of claiming R&D tax credits, and a pronounced negative impact on receiving R&D grants. In general, these results concur with those reported by Vanino et al. (2019), who report that past turnover has a limited impact on firms' likelihood of receiving public R&D support. The main exception is the case of R&D tax credits in foreign-owned firms. Here, our results seem to accord with Appelt et al. (2016), who suggest that multinational firms' decisions to use R&D tax credits in a host country may be driven by unique factors, such as reducing their global R&D cost base (relative to the use of R&D grants, and firms that do not have a foreign parent).

Table F1 below reveals that receiving other R&D policy supports (i.e. besides R&D grant/tax credit) has a strong positive impact on domestic firms' likelihood of receiving both R&D grants and R&D tax credits. In contrast, other R&D supports have no significant impact on foreign-owned firms’ likelihood of receiving either R&D grants or R&D tax credits. This result is in line with those reported by Hewitt-Dundas and Roper (2010) and Busom et al. (2014), which suggest that firms with more limited technological capabilities (likely domestic firms), may need to draw on other forms of public R&D support (i.e. beyond supports such as R&D grants and R&D tax credits). Finally, Table F1 shows that large and medium-sized foreign-owned firms are more likely to receive R&D grants, relative to smaller firms. These results concur with those reported by González and Pazó (2008) and Aristei et al. (2017), who show that the process of allocating grants often favors larger firms.

Overall, Table F1 suggests there are different factors at play when foreign-owned and domestic firms select into using R&D grants and R&D tax credits. The results from Table F1, therefore, support our decision to split the sample, and examine the impact of public R&D funding separately in each ownership type. Tables F2 and F3 below test whether our matching process achieved sufficient balance between treated and untreated firms to perform a robust analysis. The results reported in these tables show that the covariates in matched untreated and treated firms are well balanced.

| **Table F1:** Logit model for firms' probability of receiving public R&D funding | | | | | |
| --- | --- | --- | --- | --- | --- |
|  | Foreign-owned firms | |  | Domestic firms | |
| Variables | R&D grants | R&D tax credit |  | R&D grants | R&D tax credit |
| R&D above median | 1.070*** | 0.577*** |  | 0.334*** | 1.570*** |
|  | (0.140) | (0.104) |  | (0.0604) | (0.0536) |
| Past turnover quartile two | -0.661*** | 0.558*** |  | -0.200** | 0.0687 |
|  | (0.256) | (0.171) |  | (0.0892) | (0.0814) |
| Past turnover quartile three | -0.601** | 0.496*** |  | -0.194** | -0.136 |
|  | (0.268) | (0.182) |  | (0.0965) | (0.0892) |
| Past turnover quartile four | -0.266 | 0.464** |  | -0.275** | -0.186* |
|  | (0.277) | (0.198) |  | (0.120) | (0.109) |
| Previous public R&D funding | 3.600*** | 2.911*** |  | 2.484*** | 2.278*** |
|  | (0.265) | (0.121) |  | (0.0800) | (0.0611) |
| Other R&D support | 0.380** | -0.198 |  | 0.878*** | 0.539*** |
|  | (0.159) | (0.136) |  | (0.0649) | (0.0645) |
| Firm size: Small | 1.256* | 1.164*** |  | 0.0986 | 0.152* |
|  | (0.760) | (0.421) |  | (0.0865) | (0.0792) |
| Firm size: Medium | 1.722** | 1.063** |  | 0.0189 | 0.135 |
|  | (0.766) | (0.428) |  | (0.124) | (0.112) |
| Firm size: Large | 1.932** | 0.591 |  | 0.105 | -0.382** |
|  | (0.780) | (0.442) |  | (0.201) | (0.182) |
| Constant | -6.613*** | -5.579*** |  | -3.731*** | -5.116*** |
|  | (0.886) | (0.521) |  | (0.185) | (0.194) |
| Observations | 4,051 | 4,023 |  | 15,756 | 15,756 |
| Log likelihood | -1535.2 | -914.82 |  | -5485.35 | -4677.96 |
| R^2^ | 0.3073 | 0.3154 |  | 0.3536 | 0.2027 |
| **Notes:** * p < 0.1; ** p < 0.05; *** p < 0.01. Standard errors in parentheses. Dummy variables for year and NACE sector are included in the propensity score estimation, but the output is not displayed here. The base category for firm size is micro; the base category for R&D intensity quartile is zero R&D; the base category for turnover is the lowest turnover quartile. | | | | | |

| **Table F2:** Balance of the control variables after matching, foreign-owned firms | | | | | | |
| --- | --- | --- | --- | --- | --- | --- |
|  | *Treatment: R&D grant (foreign-owned firms)* | | | | | |
|  | Mean |  |  | Bias | t-test |  |
| Variable | Treated | Control |  | % | t-value | p-value |
| R&D above median | 0.64444 | 0.63519 |  | 2.1 | 0.32 | 0.752 |
| Past turnover quartile two | 0.13519 | 0.12222 |  | 3.2 | 0.64 | 0.525 |
| Past turnover quartile three | 0.20926 | 0.22222 |  | -3 | -0.52 | 0.605 |
| Past turnover quartile four | 0.57222 | 0.57407 |  | -0.4 | -0.06 | 0.951 |
| Previous public R&D funding | 0.78889 | 0.78889 |  | 0 | 0 | 1 |
| Other R&D support | 0.20741 | 0.20556 |  | 0.6 | 0.08 | 0.94 |
| Firm size: Small | 0.08148 | 0.08148 |  | 0 | 0 | 1 |
| Firm size: Medium | 0.32407 | 0.32407 |  | 0 | 0 | 1 |
| Firm size: Large | 0.59074 | 0.59074 |  | 0 | 0 | 1 |
| Ps-R2 | LR-chi2 | p>chi2 | MeanBias | MedBias | Rubin's B | Rubin's R |
| 0.001 | 0.85 | 1 | 0.3 | 0 | 5.6 | 1.02 |
| On support | Off support | | Not treated | | Total | |
| 327 | 109 |  | 4,931 |  | 5,367 |  |
|  | *Treatment: R&D tax credit (foreign-owned firms)* | | | | | |
|  | Mean |  |  | Bias | t-test |  |
| Variable | Treated | Control |  | % | t-value | p-value |
| R&D above median | 0.51397 | 0.50547 |  | 1.9 | 0.34 | 0.73 |
| Past turnover quartile two | 0.23572 | 0.2661 |  | -7 | -1.42 | 0.155 |
| Past turnover quartile three | 0.26002 | 0.23572 |  | 5.6 | 1.14 | 0.254 |
| Past turnover quartile four | 0.3949 | 0.39004 |  | 1.1 | 0.2 | 0.84 |
| Previous public R&D funding | 0.84812 | 0.84812 |  | 0 | 0 | 1 |
| Other R&D support | 0.15553 | 0.14095 |  | 4.7 | 0.83 | 0.405 |
| Firm size: Small | 0.14581 | 0.14581 |  | 0 | 0 | 1 |
| Firm size: Medium | 0.45079 | 0.45079 |  | 0 | 0 | 1 |
| Firm size: Large | 0.40219 | 0.40219 |  | 0 | 0 | 1 |
| Ps-R2 | LR-chi2 | p>chi2 | MeanBias | MedBias | Rubin's B | Rubin's R |
| 0.002 | 4.14 | 1 | 0.7 | 0 | 10 | 1.18 |
| On support | Off support | | Not treated | | Total | |
| 828 | 161 |  | 4,369 |  | 5,358 |  |
| **Notes:** Year and NACE sector variables are included in the analysis but are not presented. Columns 1 and 2 present the mean value of each control variable for firms in the treated and control groups after matching. Column 3 displays the median standard bias across all the covariates after matching. Columns 4 and 5 report the t-tests of mean values between treated and matched untreated firms. The bottom rows present diagnostic tests developed by Leuven and Sianesi (2018), and summary statistics on the matched sample. The Rubin’s B score captures the absolute standardised difference of means of a linear index of the propensity score in treated and matched non-treated groups. A Rubin’s B score of below the 25 per cent is considered reliable. The Rubin’s R score shows the ratio of treated to matched non-treated variances of the propensity score index. If this ratio is within the required range of 0.5 and 2, the samples are considered to be sufficiently balanced. Total observations falls from that reported in Table C2 due to missing values in the R&D expenditure variable. | | | | | | |

| **Table F3:** Balance of the control variables after matching, domestic firms | | | | | | |
| --- | --- | --- | --- | --- | --- | --- |
|  | *Treatment: R&D grant (domestic firms)* | | | | | |
|  | Mean |  |  | Bias | t-test |  |
| Variable | Treated | Control |  | % | t-value | p-value |
| R&D above median | 0.55057 | 0.55003 |  | 0.1 | 0.03 | 0.973 |
| Past turnover quartile two | 0.22417 | 0.22198 |  | 0.5 | 0.16 | 0.874 |
| Past turnover quartile three | 0.25424 | 0.27009 |  | -3.6 | -1.09 | 0.276 |
| Past turnover quartile four | 0.24549 | 0.24604 |  | -0.1 | -0.04 | 0.969 |
| Previous public R&D funding | 0.8573 | 0.8573 |  | 0 | 0 | 1 |
| Other R&D support | 0.25752 | 0.25697 |  | 0.1 | 0.04 | 0.97 |
| Firm size: Small | 0.55221 | 0.55221 |  | 0 | 0 | 1 |
| Firm size: Medium | 0.19574 | 0.19574 |  | 0 | 0 | 1 |
| Firm size: Large | 0.02952 | 0.02952 |  | 0 | 0 | 1 |
| Ps-R2 | LR-chi2 | p>chi2 | MeanBias | MedBias | Rubin's B | Rubin's R |
| 0.001 | 3.13 | 1 | 0.2 | 0 | 5.8 | 1.06 |
| On support | Off support | | Not treated | | Total | |
| 1,820 | 181 |  | 16,828 |  | 18,829 |  |
|  | *Treatment: R&D tax credit (domestic firms)* | | | | | |
|  | Mean |  |  | Bias | t-test |  |
| Variable | Treated | Control |  | % | t-value | p-value |
| R&D above median | 0.7104 | 0.71009 |  | 0.1 | 0.03 | 0.978 |
| Past turnover quartile two | 0.2435 | 0.24412 |  | -0.1 | -0.06 | 0.954 |
| Past turnover quartile three | 0.25371 | 0.26083 |  | -1.6 | -0.65 | 0.513 |
| Past turnover quartile four | 0.27568 | 0.26918 |  | 1.5 | 0.59 | 0.557 |
| Previous public R&D funding | 0.86108 | 0.86108 |  | 0 | 0 | 1 |
| Other R&D support | 0.21535 | 0.21194 |  | 1 | 0.33 | 0.739 |
| Firm size: Small | 0.56405 | 0.56405 |  | 0 | 0 | 1 |
| Firm size: Medium | 0.22525 | 0.22525 |  | 0 | 0 | 1 |
| Firm size: Large | 0.03342 | 0.03342 |  | 0 | 0 | 1 |
| Ps-R2 | LR-chi2 | p>chi2 | MeanBias | MedBias | Rubin's B | Rubin's R |
| 0 | 1.07 | 1 | 0.1 | 0 | 2.6 | 1.22 |
| On support | Off support | | Not treated | | Total | |
| 3,271 | 415 |  | 15,143 |  | 18,829 |  |
| **Notes:** Year and NACE sector variables are included in the analysis but are not presented. Columns 1 and 2 present the mean value of each control variable for firms in the treated and control groups after matching. Column 3 displays the median standard bias across all the covariates after matching. Columns 4 and 5 report the t-tests of mean values between treated and matched untreated firms. The bottom rows present diagnostic tests developed by Leuven and Sianesi (2018), and summary statistics on the matched sample. The Rubin’s B score captures the absolute standardised difference of means of a linear index of the propensity score in treated and matched non-treated groups. A Rubin’s B score of below the 25 per cent is considered reliable. The Rubin’s R score shows the ratio of treated to matched non-treated variances of the propensity score index. If this ratio is within the required range of 0.5 and 2, the samples are considered to be sufficiently balanced. Total observations fall from that reported in Table C2 due to missing values in the R&D expenditure variable. | | | | | | |

**Appendix G: Robustness tests**

| **Table G1:** Robustness tests of propensity score matching analysis for the input additionality of R&D supports in foreign-owned and domestic firms | | | |
| --- | --- | --- | --- |
| *Matching estimator: 1:3 nearest neighbour matching* | | | |
| Foreign-owned firms | | | |
| Treatment | Treated | Matched untreated | Difference (α) |
| R&D tax credit | 0.803 | 0.108 | 0.695*** |
| R&D grant | 1.818 | 0.486 | 1.332*** |
| Domestic firms | | | |
| Treatment | Treated | Matched untreated | Difference (α) |
| R&D tax credit | 0.194 | 0.06 | 0.134*** |
| R&D grant | 0.076 | 0.038 | 0.038*** |
|  |  |  |  |
| *Matching estimator: Kernel density matching* | | | |
| Foreign-owned firms | | | |
| Treatment | Treated | Matched untreated | Difference (α) |
| R&D tax credit | 0.799 | 0.084 | 0.715*** |
| R&D grant | 1.665 | 0.396 | 1.269*** |
| Domestic firms | | | |
| Treatment | Treated | Matched untreated | Difference (α) |
| R&D tax credit | 0.194 | 0.058 | 0.136*** |
| R&D grant | 0.075 | 0.03 | 0.045*** |
| **Notes:** * p < 0.1; ** p < 0.05; *** p < 0.01. These robustness tests examine the sensitivity of our analysis using 1:1 nearest neighbour matching (reported in Table 3 in the main paper), to a change of matching estimator. For ease of interpretation, results are expressed in millions of Euros. The α term relates to the average policy-induced R&D from each policy instrument. | | | |

| **Table G2:** Robustness tests for the link between policy-induced R&D and firm performance in foreign-owned subsidiaries and domestic firms, based on 3 nearest neighbour matching | | | | | | | |
| --- | --- | --- | --- | --- | --- | --- | --- |
|  | Foreign-owned subsidiaries | | |  | Domestic firms | | |
| Variable | Turnover (log) | GVA (log) | Exports (log) |  | Turnover (log) | GVA (log) | Exports (log) |
| Privately-funded R&D | 0.00454*** | 0.00486** | 0.00433*** |  | 0.107** | 0.0872** | 0.112*** |
|  | (0.00155) | (0.00128) | (0.00114) |  | (0.0227) | (0.0157) | (0.0203) |
| Policy-induced: R&D tax credit | 0.00702** | 0.00765** | 0.00723*** |  | 0.137** | 0.107** | 0.0883*** |
|  | (0.00313) | (0.00264) | (0.00236) |  | (0.0239) | (0.0218) | (0.0282) |
| Policy-induced: R&D grant | 0.00481** | 0.0052** | 0.00454** |  | 0.105** | 0.083** | 0.134*** |
|  | (0.00191) | (0.0023) | (0.00207) |  | (0.0236) | (0.019) | (0.0247) |
| Firm size: Small | 0.111 | -0.0196 | 0.143 |  | 0.380*** | 0.412*** | 0.346*** |
|  | (0.189) | (0.114) | (0.102) |  | (0.0256) | (0.0232) | (0.0323) |
| Firm size: Medium | 0.561*** | 0.341*** | 0.498*** |  | 0.759*** | 0.783*** | 0.672*** |
|  | (0.199) | (0.123) | (0.111) |  | (0.0358) | (0.0347) | (0.0477) |
| Firm size: Large | 0.936*** | 0.674*** | 0.877*** |  | 1.143*** | 1.198*** | 0.910*** |
|  | (0.208) | (0.135) | (0.122) |  | (0.0745) | (0.0712) | (0.0971) |
| Materials | 0.205 | -0.907*** | 0.148* |  | 0.0268 | -0.784*** | 0.0591** |
|  | (0.169) | (0.0872) | (0.0802) |  | (0.0400) | (0.0382) | (0.0231) |
| Unit labour costs | -0.0458*** | -0.0540*** | -0.0546*** |  | -0.000492 | 0.00213** | -0.000378 |
|  | (0.0132) | (0.00403) | (0.00381) |  | (0.000558) | (0.00104) | (0.000940) |
| Training | 0.0708*** | 0.0839*** | 0.0590*** |  | 0.0529*** | 0.0553*** | 0.0605*** |
|  | (0.0174) | (0.0122) | (0.0114) |  | (0.00584) | (0.00569) | (0.00785) |
| Regional R&D | 0.000186 | 0.000039 | 0.000261 |  | 0.000027 | 0.000019 | 0.000013 |
|  | (0.000404) | (0.000258) | (0.000238) |  | (0.000041) | (0.00003) | (0.000041) |
| Constant | -0.929*** | -0.160 | -0.879*** |  | -0.548*** | -0.186*** | -0.805*** |
|  | (0.217) | (0.140) | (0.126) |  | (0.0482) | (0.0399) | (0.0497) |
| Firm fixed effects | Yes | Yes | Yes |  | Yes | Yes | Yes |
| Year fixed effects | Yes | Yes | Yes |  | Yes | Yes | Yes |
| Observations | 3,600 | 3,548 | 3,439 |  | 14,168 | 13,864 | 12,387 |
| R^2^ | 0.235 | 0.192 | 0.199 |  | 0.236 | 0.180 | 0.166 |
| **Notes:** * p < 0.1; ** p < 0.05; *** p < 0.01. Robust standard errors in parentheses. The policy-induced R&D variables correspond to the α term defined in Equation (2). This table uses the findings presented in Table G1,which calculates the input additionality from R&D support using 1:3 nearest neighbour matching. The number of observations changes in each model due to missing values in the dependent variables. For ease of interpretation, the variables ‘Privately-funded R&D’, ‘Policy-induced: R&D tax credit (α)’, and ‘Policy-induced: R&D grant (α)’ have been scaled by 1 million. | | | | | | | |

| **Table G3:** Robustness tests for the link between policy-induced R&D and firm performance in foreign-owned subsidiaries and domestic firms, based on kernel density matching | | | | | | | |
| --- | --- | --- | --- | --- | --- | --- | --- |
|  | Foreign-owned subsidiaries | | |  | Domestic firms | | |
| Variable | Turnover (log) | GVA  (log) | Exports (log) |  | Turnover (log) | GVA  (log) | Exports  (log) |
| Privately-funded R&D | 0.00411*** | 0.00454*** | 0.00388*** |  | 0.103*** | 0.085*** | 0.105*** |
|  | (0.000954) | (0.0012) | (0.00107) |  | (0.0116) | (0.0155) | (0.0201) |
| Policy-induced: R&D tax credit | 0.00725*** | 0.00862*** | 0.00743*** |  | 0.126*** | 0.101*** | 0.0716*** |
|  | (0.00208) | (0.00261) | (0.00233) |  | (0.0157) | (0.0211) | (0.0272) |
| Policy-induced: R&D grant | 0.00415** | 0.00429* | 0.00386* |  | 0.100*** | 0.0815*** | 0.128*** |
|  | (0.00183) | (0.00228) | (0.00205) |  | (0.014) | (0.0186) | (0.0242) |
| Firm size: Small | 0.110 | -0.0204 | 0.142 |  | 0.381*** | 0.412*** | 0.346*** |
|  | (0.0915) | (0.114) | (0.102) |  | (0.0172) | (0.0232) | (0.0323) |
| Firm size: Medium | 0.560*** | 0.340*** | 0.497*** |  | 0.761*** | 0.785*** | 0.673*** |
|  | (0.0991) | (0.123) | (0.111) |  | (0.0258) | (0.0347) | (0.0477) |
| Firm size: Large | 0.934*** | 0.672*** | 0.875*** |  | 1.143*** | 1.198*** | 0.912*** |
|  | (0.108) | (0.135) | (0.122) |  | (0.0532) | (0.0712) | (0.0971) |
| Materials | 0.205*** | -0.908*** | 0.148* |  | 0.0267** | -0.784*** | 0.0592** |
|  | (0.0686) | (0.0872) | (0.0802) |  | (0.0130) | (0.0382) | (0.0231) |
| Unit labour costs | -0.0458*** | -0.0540*** | -0.0547*** |  | -0.000496 | 0.00212** | -0.000386 |
|  | (0.00276) | (0.00403) | (0.00381) |  | (0.000414) | (0.00104) | (0.000941) |
| Training | 0.0712*** | 0.0843*** | 0.0594*** |  | 0.0530*** | 0.0553*** | 0.0608*** |
|  | (0.00974) | (0.0122) | (0.0114) |  | (0.00423) | (0.00569) | (0.00785) |
| Regional R&D | 0.000184 | 0.000036 | 0.000259 |  | 0.000028 | 0.000019 | 0.000014 |
|  | -0.00021 | -0.00026 | -0.00024 |  | (0.000023) | (0.000030) | (0.000041) |
| Constant | -0.927*** | -0.158 | -0.878*** |  | -0.548*** | -0.185*** | -0.805*** |
|  | (0.112) | (0.140) | (0.126) |  | (0.0266) | (0.0399) | (0.0497) |
| Firm fixed effects | Yes | Yes | Yes |  | Yes | Yes | Yes |
| Year fixed effects | Yes | Yes | Yes |  | Yes | Yes | Yes |
| Observations | 3,600 | 3,548 | 3,439 |  | 14,168 | 13,864 | 12,387 |
| R^2^ | 0.235 | 0.192 | 0.199 |  | 0.236 | 0.180 | 0.166 |
| **Notes:** * p < 0.1; ** p < 0.05; *** p < 0.01. Robust standard errors in parentheses. The policy-induced R&D variables correspond to the α term defined in Equation (2). This table uses the findings presented in Table G1, which calculates the input additionality from R&D support using Kernel density matching. The number of observations changes in each model due to missing values in the dependent variables. For ease of interpretation, the variables ‘Privately-funded R&D’, ‘Policy-induced: R&D tax credit (α)’, and ‘Policy-induced: R&D grant (α)’ have been scaled by 1 million. | | | | | | | |

| **Table G4:** Robustness tests for the link between policy-induced R&D from policy instrument mix and firm performance | | | | | | | |
| --- | --- | --- | --- | --- | --- | --- | --- |
|  | Foreign-owned subsidiaries | | |  | Domestic firms | | |
| Variable | Turnover (log) | GVA (log) | Exports (log) |  | Turnover (log) | GVA (log) | Exports (log) |
| Privately-funded R&D | 0.00789** | 0.00741*** | 0.00717** |  | 0.151*** | 0.129*** | 0.160*** |
|  | (0.00318) | (0.00258) | (0.00331) |  | (0.0349) | (0.0347) | (0.0389) |
| Policy-induced: R&D tax credit (no R&D grant) | 0.00712** | '0.00514* | 0.00639* |  | 0.159*** | 0.139*** | 0.158*** |
|  | (0.00324) | (0.00317) | (0.00337) |  | (0.0338) | (0.0347) | (0.0387) |
| Policy-induced: R&D tax credit (+ R&D grant) | 0.00611* | 0.00566* | 0.00552* |  | 0.159*** | 0.135*** | 0.124*** |
|  | (0.00312) | (0.00339) | (0.00318) |  | (0.0308) | (0.0322) | (0.0382) |
| Policy-induced: R&D grant (no R&D tax credit) | 0.00628* | 0.00420 | 0.00550 |  | 0.181*** | 0.136*** | 0.185*** |
|  | (0.00350) | (0.00314) | (0.00360) |  | (0.0444) | (0.0504) | (0.0650) |
| Policy-induced: R&D grant (+ R&D tax credit) | 0.00831** | 0.00834** | 0.00774** |  | 0.143*** | 0.117*** | 0.171*** |
|  | (0.00358) | (0.00347) | (0.00374) |  | (0.0354) | (0.0354) | (0.0396) |
| Firm size: Small | 0.113 | -0.0183 | 0.145 |  | 0.381*** | 0.413*** | 0.346*** |
|  | (0.188) | (0.169) | (0.190) |  | (0.0255) | (0.0296) | (0.0437) |
| Firm size: Medium | 0.564*** | 0.344* | 0.500** |  | 0.752*** | 0.776*** | 0.664*** |
|  | (0.199) | (0.185) | (0.199) |  | (0.0359) | (0.0425) | (0.0638) |
| Firm size: Large | 0.935*** | 0.671*** | 0.875*** |  | 1.130*** | 1.184*** | 0.892*** |
|  | (0.208) | (0.199) | (0.217) |  | (0.0753) | (0.0888) | (0.143) |
| Materials | 0.199 | -0.912*** | 0.144 |  | 0.0253 | -0.788*** | 0.0579*** |
|  | (0.170) | (0.182) | (0.158) |  | (0.0409) | (0.0787) | (0.0223) |
| Unit labour costs | -0.0454*** | -0.0535*** | -0.0539*** |  | -0.000532 | 0.00211 | -0.000400 |
|  | (0.0133) | (0.0166) | (0.0168) |  | (0.000561) | (0.00175) | (0.000977) |
| Training | 0.0697*** | 0.0822*** | 0.0581*** |  | 0.0518*** | 0.0542*** | 0.0590*** |
|  | (0.0174) | (0.0202) | (0.0181) |  | (0.00586) | (0.00737) | (0.0102) |
| Regional R&D | 0.0141 | 0.0628 | 0.0234 |  | 0.0158 | -0.00135 | -0.0208 |
|  | (0.0418) | (0.0538) | (0.0467) |  | (0.0194) | (0.0262) | (0.0407) |
| Constant | 16.06*** | 15.07*** | 15.74*** |  | 14.12*** | 14.02*** | 13.33*** |
|  | (0.781) | (0.992) | (0.872) |  | (0.357) | (0.482) | (0.746) |
| Firm fixed effects | Yes | Yes | Yes |  | Yes | Yes | Yes |
| Year fixed effects | Yes | Yes | Yes |  | Yes | Yes | Yes |
| Observations | 3,600 | 3,548 | 3,439 |  | 14,168 | 13,864 | 12,387 |
| R^2^ | 0.236 | 0.192 | 0.199 |  | 0.238 | 0.181 | 0.167 |
| **Notes:** * p < 0.1; ** p < 0.05; *** p < 0.01. Robust standard errors in parentheses. The policy-induced R&D variables correspond to the α term defined in Equation (2). The number of observations changes in each model due to missing values in the dependent variables. For ease of interpretation, the variable ‘Privately-funded R&D’, and all of the ‘Policy-induced: R&D’ variables have been scaled by 1 million. | | | | | | | |

| **Table G5:** Robustness tests for the link between policy-induced R&D from policy instrument mix and firm performance, based on 1:3 nearest neighbour matching | | | | | | | |
| --- | --- | --- | --- | --- | --- | --- | --- |
|  | Foreign-owned subsidiaries | | |  | Domestic firms | | |
| Variable | Turnover (log) | GVA (log) | Exports (log) | | Turnover (log) | GVA (log) | Exports (log) |
| Privately-funded R&D | 0.00445*** | 0.00463** | 0.00424** | | 0.106*** | 0.0864*** | 0.113*** |
|  | (0.00156) | (0.0018) | (0.0017) |  | (0.0225) | (0.0237) | (0.0256) |
| Policy-induced: R&D tax credit (no R&D grant) | 0.00648** | 0.0054 | 0.00629** | | 0.152*** | 0.11** | 0.122*** |
|  | (0.00292) | (0.00336) | (0.0032) |  | (0.0362) | (0.0428) | (0.0385) |
| Policy-induced: R&D tax credit (+ R&D grant) | 0.0063** | 0.00693** | 0.00664* |  | 0.134*** | 0.107*** | 0.0793** |
|  | (0.00312) | (0.00286) | (0.00343) |  | (0.0235) | (0.0288) | (0.0351) |
| Policy-induced: R&D grant (no R&D tax credit) | 0.00245 | 0.00172 | 0.00227 |  | 0.164*** | 0.122** | 0.113* |
|  | (0.00225) | (0.00246) | (0.00232) |  | (0.0456) | (0.0529) | (0.0669) |
| Policy-induced: R&D grant (+ R&D tax credit) | 0.00644*** | 0.00856*** | 0.00636*** | | 0.0998*** | 0.0803*** | 0.133*** |
|  | (0.00235) | (0.00261) | (0.00245) |  | (0.0231) | (0.0241) | (0.0266) |
| Firm size: Small | 0.111 | -0.0198 | 0.143 |  | 0.382*** | 0.413*** | 0.347*** |
|  | (0.189) | (0.170) | (0.190) |  | (0.0255) | (0.0296) | (0.0437) |
| Firm size: Medium | 0.561*** | 0.342* | 0.498** |  | 0.762*** | 0.785*** | 0.673*** |
|  | (0.199) | (0.186) | (0.199) |  | (0.0357) | (0.0424) | (0.0638) |
| Firm size: Large | 0.934*** | 0.670*** | 0.874*** |  | 1.145*** | 1.199*** | 0.909*** |
|  | (0.208) | (0.199) | (0.217) |  | (0.0746) | (0.0880) | (0.143) |
| Materials | 0.203 | -0.908*** | 0.148 |  | 0.0264 | -0.784*** | 0.0589*** |
|  | (0.169) | (0.181) | (0.157) |  | (0.0402) | (0.0786) | (0.0217) |
| Unit labour costs | -0.0457*** | -0.0540*** | -0.0545*** | | -0.000499 | 0.00212 | -0.000380 |
|  | (0.0133) | (0.0163) | (0.0165) |  | (0.000563) | (0.00175) | (0.000977) |
| Training | 0.0707*** | 0.0828*** | 0.0589*** |  | 0.0530*** | 0.0554*** | 0.0606*** |
|  | (0.0174) | (0.0201) | (0.0181) |  | (0.00585) | (0.00735) | (0.0102) |
| Regional R&D | 0.0212 | 0.0671 | 0.0288 |  | 0.0171 | -0.000322 | -0.0189 |
|  | (0.0412) | (0.0537) | (0.0464) |  | (0.0194) | (0.0262) | (0.0406) |
| Constant | 15.94*** | 15.00*** | 15.65*** |  | 14.10*** | 14.00*** | 13.30*** |
|  | (0.773) | (0.987) | (0.869) |  | (0.357) | (0.482) | (0.745) |
| Firm fixed effects | Yes | Yes | Yes |  | Yes | Yes | Yes |
| Year fixed effects | Yes | Yes | Yes |  | Yes | Yes | Yes |
| Observations | 3,600 | 3,548 | 3,439 |  | 14,168 | 13,864 | 12,387 |
| R^2^ | 0.235 | 0.193 | 0.199 |  | 0.236 | 0.180 | 0.166 |
| **Notes:** * p < 0.1; ** p < 0.05; *** p < 0.01. Robust standard errors in parentheses. The policy-induced R&D variables correspond to the α term defined in Equation (2). This table uses the findings presented in Table G1, which calculates the input additionality from R&D support using 1:3 nearest neighbour matching. The number of observations changes in each model due to missing values in the dependent variables. For ease of interpretation, the variable ‘Privately-funded R&D’, and all of the ‘Policy-induced: R&D’ variables have been scaled by 1 million. | | | | | | | |

| **Table G6:** Robustness tests for the link between policy-induced R&D from policy instrument mix and firm performance, based on kernel density matching | | | | | | | |
| --- | --- | --- | --- | --- | --- | --- | --- |
|  | Foreign-owned subsidiaries | | |  | Domestic firms | | |
| Variable | Turnover (log) | GVA  (log) | Exports  (log) | | Turnover (log) | GVA (log) | Exports (log) |
| Privately-funded R&D | 0.00404*** | 0.00432** | 0.0038** |  | 0.101*** | 0.0842*** | 0.106*** |
|  | (0.00137) | (0.00173) | (0.0015) |  | (0.0219) | (0.0232) | (0.0248) |
| Policy-induced: R&D tax credit (no R&D grant) | 0.00669** | 0.00624* | 0.0065* |  | 0.148*** | 0.111*** | 0.11*** |
|  | (0.00302) | (0.0036) | (0.00338) |  | (0.0347) | (0.0413) | (0.0367) |
| Policy-induced: R&D tax credit (+ R&D grant) | 0.00643* | 0.00771** | 0.00673* |  | 0.122*** | 0.101*** | 0.0626* |
|  | (0.00335) | (0.00337) | (0.00384) |  | (0.0223) | (0.0267) | (0.032) |
| Policy-induced: R&D grant (no R&D tax credit) | 0.00193 | 0.000916 | 0.00172 |  | 0.152*** | 0.128*** | 0.103* |
|  | (0.00212) | (0.00252) | (0.00217) |  | (0.0427) | (0.0491) | (0.0623) |
| Policy-induced: R&D grant (+ R&D tax credit) | 0.0058*** | 0.00786*** | 0.00569*** | | 0.0949*** | 0.0776*** | 0.127*** |
|  | (0.00213) | (0.00234) | (0.00219) |  | (0.0222) | (0.0234) | (0.0255) |
| Firm size: Small | 0.110 | -0.0205 | 0.142 |  | 0.382*** | 0.413*** | 0.347*** |
|  | (0.189) | (0.170) | (0.190) |  | (0.0255) | (0.0296) | (0.0437) |
| Firm size: Medium | 0.560*** | 0.341* | 0.497** |  | 0.763*** | 0.786*** | 0.674*** |
|  | (0.199) | (0.186) | (0.199) |  | (0.0357) | (0.0424) | (0.0639) |
| Firm size: Large | 0.932*** | 0.669*** | 0.872*** |  | 1.145*** | 1.199*** | 0.911*** |
|  | (0.208) | (0.199) | (0.217) |  | (0.0748) | (0.0881) | (0.143) |
| Materials | 0.202 | -0.909*** | 0.147 |  | 0.0263 | -0.784*** | 0.0589*** |
|  | (0.170) | (0.181) | (0.158) |  | (0.0402) | (0.0786) | (0.0217) |
| Unit labour costs | -0.0457*** | -0.0540*** | -0.0545*** | | -0.000504 | 0.00211 | -0.000389 |
|  | (0.0133) | (0.0163) | (0.0165) |  | (0.000565) | (0.00174) | (0.000979) |
| Training | 0.0710*** | 0.0831*** | 0.0593*** |  | 0.0531*** | 0.0554*** | 0.0609*** |
|  | (0.0174) | (0.0201) | (0.0181) |  | (0.00587) | (0.00736) | (0.0102) |
| Regional R&D | 0.0224 | 0.0677 | 0.0301 |  | 0.0172 | -0.000283 | -0.0187 |
|  | (0.0411) | (0.0534) | (0.0464) |  | (0.0194) | (0.0262) | (0.0406) |
| Constant | 15.91*** | 14.99*** | 15.62*** |  | 14.10*** | 14.00*** | 13.29*** |
|  | (0.773) | (0.984) | (0.870) |  | (0.357) | (0.481) | (0.744) |
| Firm fixed effects | Yes | Yes | Yes |  | Yes | Yes | Yes |
| Year fixed effects | Yes | Yes | Yes |  | Yes | Yes | Yes |
| Observations | 3,600 | 3,548 | 3,439 |  | 14,168 | 13,864 | 12,387 |
| R^2^ | 0.236 | 0.194 | 0.199 |  | 0.236 | 0.180 | 0.166 |
| **Notes:** * p < 0.1; ** p < 0.05; *** p < 0.01. Robust standard errors in parentheses. The policy-induced R&D variables correspond to the α term defined in Equation (2). This table uses the findings presented in Table G1, which calculates the input additionality from R&D support using Kernel density matching. The number of observations changes in each model due to missing values in the dependent variables. For ease of interpretation, the variable ‘Privately-funded R&D’, and all of the ‘Policy-induced: R&D’ variables have been scaled by 1 million. | | | | | | | |

| **Table G7:** Robustness tests for the link between policy-induced R&D and firm performance in foreign-owned subsidiaries and domestic firms, based on the Lewbel instrumental variable model | | | | | | | |
| --- | --- | --- | --- | --- | --- | --- | --- |
|  | Foreign-owned subsidiaries | | |  | Domestic firms | | |
| Variable | Turnover (log) | GVA  (log) | Exports  (log) | | Turnover  (log) | GVA  (log) | Exports  (log) |
| Privately-funded R&D | 0.0141*** | 0.0797* | 0.0152*** |  | 0.176*** | 0.097*** | 0.175*** |
|  | (0.00350) | (0.00449) | (0.00454) |  | (0.0272) | (0.0366) | (0.0474) |
| Policy-induced: R&D tax credit | 0.0131*** | 0.00737* | 0.0145*** |  | 0.167*** | 0.1063*** | 0.170*** |
|  | (0.00379) | (0.0039) | (0.00548) |  | (0.0277) | (0.0377) | (0.0483) |
| Policy-induced: R&D grant | 0.0111*** | 0.000625 | 0.01368*** |  | 0.169*** | 0.0964** | 0.195*** |
|  | (0.0038) | (0.00497) | (0.00490) |  | (0.0288) | (0.0388) | (0.0504) |
| Firm size: Small | 0.101 | -0.0262 | 0.134 |  | 0.379*** | 0.413*** | 0.346*** |
|  | (0.0914) | (0.114) | (0.102) |  | (0.0171) | (0.0232) | (0.0323) |
| Firm size: Medium | 0.552*** | 0.336*** | 0.480*** |  | 0.747*** | 0.780*** | 0.663*** |
|  | (0.0990) | (0.123) | (0.112) |  | (0.0259) | (0.0350) | (0.0480) |
| Firm size: Large | 0.920*** | 0.663*** | 0.846*** |  | 1.126*** | 1.194*** | 0.889*** |
|  | (0.108) | (0.135) | (0.122) |  | (0.0535) | (0.0721) | (0.0981) |
| Materials | 0.184*** | -0.912*** | 0.133 |  | 0.0277** | -0.778*** | 0.0599*** |
|  | (0.0699) | (0.0879) | (0.0810) |  | (0.0129) | (0.0381) | (0.0230) |
| Unit labour costs | -0.0453*** | -0.0533*** | -0.0540*** | | -0.000500 | 0.00211** | -0.000313 |
|  | (0.00276) | (0.00403) | (0.00379) |  | (0.000411) | (0.00103) | (0.000937) |
| Training | 0.0708*** | 0.0837*** | 0.0589*** |  | 0.0522*** | 0.0559*** | 0.0598*** |
|  | (0.0097) | (0.0122) | (0.0114) |  | (0.00425) | (0.00575) | (0.00792) |
| Regional R&D | 0.00729 | -0.06759 | 0.01552 |  | 0.01751 | 0.00095 | -0.01707 |
|  | (0.0326) | (0.0409) | (0.03694) | | (0.01529) | (0.0206) | (0.02830) |
| Firm fixed effects | Yes | Yes | Yes |  | Yes | Yes | Yes |
| Year fixed effects | Yes | Yes | Yes |  | Yes | Yes | Yes |
| Observations | 3,600 | 3,548 | 3,439 |  | 14,168 | 13,864 | 12,387 |
| R^2^ | 0.233 | 0.186 | 0.195 |  | 0.246 | 0.182 | 0.170 |
| Over-identification test of all instruments (Hansen J statistic) | 20.719 | 26.062 | 25.731 |  | 23.730 | 31.535 | 19.060 |
| Hansen J statistic p*-*value | 0.9993 | 0.9893 | 0.9907 |  | 0.9977 | 0.9358 | 0.9998 |
| Under-identification test (Kleibergen-Paap rk LM statistic) | 34.439 | 33.851 | 31.35 |  | 43.876 | 41.095 | 42.015 |
| Kleibergen-Paap rk LM statistic p-value | 0.8949 | 0.9079 | 0.9512 |  | 0.5616 | 0.6774 | 0.6399 |
| Kleibergen-Paap rk Wald F | 16.393 | 14.875 | 11.657 |  | 68.693 | 66.198 | 60.437 |
| **Notes:** * p < 0.1; ** p < 0.05; *** p < 0.01. Robust standard errors in parentheses. Instrumental variable method based on Lewbel (2012). No constant term is included in the output, because fixed effects are used. The policy-induced R&D variables correspond to the α term defined in Equation (2). The number of observations changes in each model due to missing values in the dependent variables. The variables ‘Privately-funded R&D’, ‘Policy-induced: R&D tax credit’, and ‘Policy-induced: R&D grant’ were instrumented by the following variables: Firm size (small, medium, and large), year dummy variables, materials, unit labour costs, training, and regional R&D. For ease of interpretation, the variables ‘Privately-funded R&D’, ‘Policy-induced: R&D tax credit’, and ‘Policy-induced: R&D grant’ have been scaled by 1 million. The statistical tests in the final five rows of Table G6 can be interpreted as follows: The Hansen J statistic developed by Hansen (1982) is a test of over-identification of restrictions in the model. The null hypothesis is that the over-identification restrictions of the model are valid (i.e. instruments are uncorrelated with the error terms). The bottom two rows present tests developed by Kleibergen and Paap (2006). The Kleibergen-Paap rk LM statistic is a test of under-identification, which indicates whether the excluded instruments are correlated with the endogenous regressors (i.e. relevance of instruments). The Kleibergen-Paap rk Wald F is a test for weak instruments, which measures weak correlation between the instruments and the regressors. The null hypothesis is that instruments are weakly correlated. | | | | | | | |

**Reference list**

Aerts, K. (2008). Carrying Flemish coals to Newcastle? R&D subsidies and foreign ownership. In K. Aerts, & D. Czarnitzki (Eds.), *Essays on the Economics of Evaluation: Public Policy and Corporate Strategies in Innovation* (pp. 127-167). Belgium: Katholieke Universiteit Leuven.

Allison, P. D., Williams, R., & Moral-Benito, E. (2017). Maximum likelihood for cross-lagged panel models with fixed effects. *Socius*, 3, 1-17. <https://doi.org/10.1177/2378023117710578>

Appelt, S., Bajgar, M., Criscuolo, F., & Galindo-Rueda, C. (2016). R&D Tax Incentives: Evidence on design, incidence and impacts. *OECD Science, Technology and Industry Policy Papers*, No. 32. <http://dx.doi.org/10.1787/5jlr8fldqk7j-en>

Aristei, D., Vecchi, M., & Venturini, F. (2016). University and inter-firm R&D collaborations: propensity and intensity of cooperation in Europe. *The Journal of Technology Transfer*, *41*(4), 841-871. <https://doi.org/10.1007/s10961-015-9403-1>

Bailey, D., & Lenihan, H. (2015). A critical reflection on Irish industrial policy: a strategic choice approach. *International Journal of the Economics of Business*, *22*(1), 47-71. <https://doi.org/10.1080/13571516.2014.993218>

Belitz, H., & Mölders, F. (2016). International knowledge spillovers through high-tech imports and R&D of foreign-owned firms. *The Journal of International Trade & Economic Development*, *25*(4), 590-613. <https://doi.org/10.1080/09638199.2015.1106575>

Busom, I., Corchuelo, B., & Martínez-Ros, E. (2014). Tax incentives… or subsidies for business R&D?. *Small Business Economics*, 43(3), 571-596. <https://doi.org/10.1007/s11187-014-9569-1>

Cin, B. C., Kim, Y. J., & Vonortas, N.S. (2017). The impact of public R&D subsidy on small firm productivity: evidence from Korean SMEs. *Small Business Economics*, *48*(2), 345-360. <https://doi.org/10.1007/s11187-016-9786-x>

Cunningham, J.A., & Golden, W. (2015). National innovation system of Ireland. *Wiley Encyclopedia of Management*, 1–14. <https://doi.org/10.1002/9781118785317.weom130051>

Cunningham, J.A., Collins, P., & Giblin, M. (2020). Evolution of Ireland’s Industrial, Science and Technology Policy. *Annals of Science and Technology Policy*, *4*(2), 80-210. <http://dx.doi.org/10.1561/110.00000013>

Cunningham, J.A., & Link, A.N. (2021). *Technology and Innovation Policy: An International Perspective*. Edward Elgar Publishing.

Czarnitzki, D., & Lopes-Bento, C. (2013). Value for money? New microeconometric evidence on public R&D grants in Flanders. *Research Policy*, *42*(1), 76-89. <https://doi.org/10.1016/j.respol.2012.04.008>

Department of Business, Enterprise and Innovation (DJEI; 2014a), *Policy Statement on Foreign Direct Investment in Ireland*. Dublin: Department of Business, Enterprise and Innovation.

Department of Further and Higher Education, Research, Innovation and Science (DFHERIS; 2022). *Impact 2030: Ireland’s Research and Innovation Strategy*. DFHERIS: Dublin.

DJEI (2014b). *Evaluation of enterprise supports for research development and innovation*. DJEI: Dublin.

DBEI (2018). *Annual Business Survey of Economic Impact: Survey Information Booklet*. Dublin: Department of Business, Enterprise and Innovation.

European Commission (2017a). *Internationalisation of business investments in research and development and analysis of their economic impact (BERD Flows)*. Luxembourg: Publications Office of the European Union.

European Commission (2017b). *R&D tax incentives: How to make them most effective?* Luxembourg: Publications Office of the European Union. <https://data.europa.eu/doi/10.2777/976217>

European Commission (2007). Internationalisation of R&D – Facing the Challenge of Globalisation: Approaches to a Proactive International Policy in S&T. CREST Working Group: Brussels. <https://ec.europa.eu/invest-in-research/pdf/download_en/report_international.pdf>

Freel, M., Liu, R., & Rammer, C. (2019). The export additionality of innovation policy. *Industrial and Corporate Change*, *28*(5), 1257-1277. <https://doi.org/10.1093/icc/dty059>

González, X., & Pazó, C. (2008). Do public subsidies stimulate private R&D spending?. *Research Policy*, *37*(3), 371-389. <https://doi.org/10.1016/j.respol.2007.10.009>

Hansen, L.P. (1982). Large sample properties of generalized method of moments estimators. *Econometrica: Journal of the Econometric Society*, 1029-1054.

Hewitt-Dundas, N., & Roper, S. (2010). Output additionality of public support for innovation: evidence for Irish manufacturing plants. *European Planning Studies*, 18(1), 107-122. <https://doi.org/10.1080/09654310903343559>

Hud, M., & Hussinger, K. (2015). The impact of R&D subsidies during the crisis. *Research Policy,* 44(10), 1844-1855. <https://doi.org/10.1016/j.respol.2015.06.003>

Kleibergen, F., & Paap, R. (2006). Generalized Reduced Rank Tests Using the Singular Value Decomposition. *Journal of Econometrics*, *133*(1), 97–126.

Lenihan, H., Mulligan, K., & O’Driscoll, J. (2020). *A cross-country repository of details on the innovation and science policy instruments available to firms in eight countries (2007-2020): The devil is in the detail*. Kemmy Business School: University of Limerick. <http://hdl.handle.net/10344/9543>

Leuven, E., & Sianesi, B. (2018). PSMATCH2: Stata module to perform full Mahalanobis and propensity score matching, common support graphing, and covariate imbalance testing. *Statistical Software Components from Boston College Department of Economics*.

Lewbel, A. (2012). Using heteroscedasticity to identify and estimate mismeasured and endogenous regressor models. *Journal of Business & Economic Statistics*, *30*(1), 67-80. [https://doi.org/10.1080
/07350015.2012.643126](https://doi.org/10.1080/07350015.2012.643126)

OECD (2018), *OECD Economic Surveys – Ireland*. Paris: OECD Publishing.

OECD (2020). FDI Qualities Assessment of Ireland. OECD: Paris.
[https://www.oecd.org
/daf/inv/investment-policy/FDI-Qualities-Assessment-of-Ireland.pdf](https://www.oecd.org/daf/inv/investment-policy/FDI-Qualities-Assessment-of-Ireland.pdf)

Papanastassiou, M., Pearce, R., & Zanfei, A. (2020). Changing perspectives on the internationalization of R&D and innovation by multinational enterprises: A review of the literature. *Journal of International Business Studies*, *51*(4), 623-664. <https://doi.org/10.1057/s41267-019-00258-0>

Vanino, E., Roper, S., & Becker, B. (2019). Knowledge to money: Assessing the business performance effects of publicly-funded R&D grants. *Research Policy*, *48*(7), 1714-1737. <https://doi.org/10.1016/j.respol.2019.04.001>

1. See: <https://www.cso.ie/en/releasesandpublications/ep/p-fdi/foreigndirectinvestmentinireland2019/>. [↑](#footnote-ref-2)
2. See: <https://www.cso.ie/en/releasesandpublications/er/berd/businessexpenditureonresearchdevelopment2017-2018/>. [↑](#footnote-ref-3)
3. See Appendix Table E1 below, which provides representativeness tests for our sample against the full IDA data. Appendix Tables E2 and E3 below provide representativeness tests for the Enterprise Ireland and R&D tax credit data, respectively. [↑](#footnote-ref-4)
4. From 2007-2010 and also in 2016, the Irish government implemented a knowledge development box. In contrast to R&D tax credits, which are cost-based, knowledge development boxes are income-based. They function through tax deduction on income earned from qualifying assets such as patents or intellectual property. However, no data is available to our study on whether firms availed of the knowledge development box, and it was a relatively small policy instrument in the Irish context, with only 10 firms availing of it in 2016 (see: <https://data.oireachtas.ie/ie/oireachtas/committee/dail/32/committee_of_public_accounts/reports/2018/2018-06-13_examination-of-matters-in-relation-to-receipts-from-corporation-tax_en.pdf>). [↑](#footnote-ref-5)
5. See: <https://www.revenue.ie/en/companies-and-charities/reliefs-and-exemptions/research-and-development-rd-tax-credit/index.aspx>. [↑](#footnote-ref-6)
6. For more information, see: [https://www.enterprise-ireland.com/en/Research-Innovation/Companies/
   Collaborate-with-companies-research-institutes/](https://www.enterprise-ireland.com/en/Research-Innovation/Companies/Collaborate-with-companies-research-institutes/). [↑](#footnote-ref-7)
7. For more information, see: <https://www.sfi.ie/sfi-research-centres/>. [↑](#footnote-ref-8)
8. See page 1 of the *Guidelines for completing an application for IDA Ireland RD&I grant support*: <https://www.idaireland.com/getmedia/d3727e88-bb50-4efb-bbbc-cf121321ddb3/RDI-Grant-Application-Form-Guidelines-(v1-1).pdf> [↑](#footnote-ref-9)
